# Where are we in the implementation of tissue-specific epigenetic clocks?

**DOI:** 10.3389/fbinf.2024.1306244

**Published:** 2024-03-04

**Authors:** Claudia Sala, Pietro Di Lena, Danielle Fernandes Durso, Italo Faria do Valle, Maria Giulia Bacalini, Daniele Dall’Olio, Claudio Franceschi, Gastone Castellani, Paolo Garagnani, Christine Nardini

**Affiliations:** ^1^ Department of Medical and Surgical Sciences, University of Bologna, Bologna, Italy; ^2^ Department of Computer Science and Engineering, University of Bologna, Bologna, Italy; ^3^ National Counsel of Technological and Scientific Development (CNPq), Ministry of Science Technology and Innovation (MCTI), Brasília, Brazil; ^4^ CAPES Foundation, Ministry of Education of Brazil, Brasília, Brazil; ^5^ IRCCS Istituto delle Scienze Neurologiche di Bologna, Bologna, Italy; ^6^ Institute of Information Technologies, Mathematics and Mechanics, Lobachevsky University, Nizhny Novgorod, Russia; ^7^ Istituto per le Applicazioni del Calcolo “Mauro Picone”, Consiglio Nazionale delle Ricerche, Roma, Italy

**Keywords:** methylage, DNA methylation, aging, biological age, epigenetic clock, Illumina HumanMethylation450 BeadChip

## Abstract

**Introduction:** DNA methylation clocks presents advantageous characteristics with respect to the ambitious goal of identifying very early markers of disease, based on the concept that accelerated ageing is a reliable predictor in this sense.

**Methods:** Such tools, being epigenomic based, are expected to be conditioned by sex and tissue specificities, and this work is about quantifying this dependency as well as that from the regression model and the size of the training set.

**Results:** Our quantitative results indicate that elastic-net penalization is the best performing strategy, and better so when—unsurprisingly—the data set is bigger; sex does not appear to condition clocks performances and tissue specific clocks appear to perform better than generic blood clocks. Finally, when considering all trained clocks, we identified a subset of genes that, to the best of our knowledge, have not been presented yet and might deserve further investigation: CPT1A, MMP15, SHROOM3, SLIT3, and SYNGR.

**Conclusion:** These factual starting points can be useful for the future medical translation of clocks and in particular in the debate between multi-tissue clocks, generally trained on a large majority of blood samples, and tissue-specific clocks.

## 1 Introduction

DNA methylation clocks are powerful tools in translational medicine as they carry the potential to estimate biological age (methylage) and predict the risk of early-onset frailty and mortality (Oblak et al., 2021). Indeed, the ability to identify markers of accelerated aging carries the potential to develop strategies to prevent non-communicable age-related diseases (NCDs), whose burden worldwide has been by now defined as a silent pandemic (Saha and Alleyne, 2018). In particular, cancer being among the most deadly NCDs, research studies on epigenetic clocks have very often concentrated on the early identification of persons at high risk of cancer development and on the stratification of subtypes of oncological patients with different prognoses (Zheng et al., 2016; Durso et al., 2017a; Durso et al., 2017b; Johnstone et al., 2022). From the pioneering work of Hannum et al. (2013), Horvath (2013), and Weidner et al. (2014), numerous new clocks (see EstimAge for a recent summary (Di Lena et al., 2021)) and variants (Levine et al., 2018; Lu et al., 2019; Belsky et al., 2020) have been designed. In the translational perspective, the most appealing approach relies on minimally invasive one-catch-all blood clocks, representing the holy grail of cost-effective prevention, owing to the limited invasiveness (blood drawn) and savings associated with effective prevention. However, with the epigenome being among the most specific cell-defining features, it is to be expected that tissue-specific clocks may better express divergences from physiology. Despite their added invasiveness and risks (pain, bleeding, and infection), understanding in detail the differential performances of the two (blood versus tissue-specific) approaches offers an important insight into the real potential of epigenetic clocks and the bases for the construction of more efficient tools. Focusing on the ability of epigenetic clocks to provide methylage estimates that are correlated with chronological age in healthy subjects, we offer a broad analysis of the dependency of the performance of epigenetic clocks on a number of relevant variables, including regression model, size of the training set, sex, and sample tissue of origin. Our work builds on existing research aiming at the exploitation of methylage as a predictor of frailty, lifespan, and other clinically relevant parameters and intends to assess the impact of covariates that may hamper the real comparability of such translational performances. These variables are parameters by which we assess the clock performance and include the regression model, the size of the training set, the impact of sex, the tissue specificity, and in particular, given the expectations toward these techniques, the differential performance of tissue-specific and blood-specific tissues. This latter analysis is, in fact, deemed relevant to gain a clear understanding of the performance of the so-called multi-tissue clocks. Indeed, despite their name, multi-tissue clocks are, in general, trained on a very large majority of blood samples, thus leaving so far the actual predictive power of such *universal* models unclear, which might be erroneously interpreted as derived from multiple tissues. Our results represent a solid foundation to quantify the clock performance at the state-of-the-art in this continuously active research field, potentially paving the way for the development of better performing epigenetic clocks.

## 2 Materials and methods

### 2.1 Dataset selection

In order to implement tissue-specific methylage clocks, we retrieved all the DNA methylation datasets previously used by Di Lena et al. (2020). These include all the DNA methylation datasets obtained using the Illumina HumanMethylation450 BeadChip (platform GPL13534) for which *β*-values are available on the NCBI database Gene Expression Omnibus (Edgar et al., 2002). The list of datasets was updated on 12 December 2020. All datasets were carefully filtered according to the following criteria using a combination of automatic and manual curation to ensure that samples from healthy subjects would be selected (for instance, excluding normal tissues adjacent to cancer tissues from oncologic patients): only datasets for which tissue and subject age, sex, and categorization based on health status (control or case) were available were retained; samples from subjects younger than (≤) 20 years or older than (>) 85 years were removed due to the fact that the relation between methylage and chronological age is known to be not linear in those age ranges (Horvath, 2013; El Khoury et al., 2019). This implies that fetal and placental samples were also discarded; moreover, when paired samples (twins, subjects from the same family, and repeated measurements on the same sample or subject) were present, only one randomly selected sample was used in the analysis. All tissues for which DNA methylation samples were available were coded and hierarchically classified based on the Medical Subject Headings (MeSH) vocabulary (Lipscomb, 2000). The list of the datasets can be found in Supplementary Table S1.

### 2.2 Data pre-processing

First, for each dataset, we imputed the missing data with *metyhLImp* (Di Lena et al., 2019). Then, we selected a reference dataset for each tissue, and we used *regRCPqn* (Sala et al., 2020) to normalize the data. Within each tissue, before proceeding with the training of the model, we selected only the CpGs present both in the training and validation sets, and we filtered out the remaining CpGs with missing values, as well as the unreliable CpGs, as defined by Zhou et al. (2017), the rs (SNP) control probes, and all CpGs on the X and Y chromosomes. The final number of CpGs used to train each clock is given in Supplementary Table S1. Finally, we converted the processed *β*-values into M-values using the logit2 transformation (M-value = *log*_2_(*β*/(1 − *β*))) because of their more desirable statistical properties (Du et al., 2010).

### 2.3 Modeling strategy

We obtained tissue-specific predictive models of methylage considering three possible types of penalized linear regressions: ridge (L2 penalization), lasso (L1 penalization), and elastic-net [equally weighted L1 and L2 penalizations as in the studies by Hannum et al. (2013); Horvath (2013); Horvath et al. (2018); Levine et al. (2018); Zhang et al. (2019); Lee et al. (2020); Shireby et al. (2020); and Voisin et al. (2020)]. Model fitting and prediction were performed using the *cv*.*glmnet* function of the *glmnet* library v4.1.2 in R v4.2.2 and selecting the penalty parameter that minimized the mean-squared error. Each model was trained using the default parameters but with nlambda = 50 rather than 100 to lower the RAM usage. Moreover, an internal 10-fold cross-validation was performed when the number of samples was at least 10, while a leave-one-out cross-validation was performed when the number of samples was lower than 10.

To assess the predictive accuracy of the models on external datasets, a further leave-one-out cross-validation in which each dataset is iteratively left out from the training set was performed within each tissue. It should be noted that a clock was only computed when there were at least three samples in both the training set and validation set. In order to test how the model performance changes with the number of samples in the training set, we performed a stratified subsampling (by age) of the training set of each clock considering various possible dimensions, i.e., 25; 50; 75; 100; 125; 150; 175; 200; 300; 400; 500; 750; 1,000; 1,250; 1,750; 2,000; 2,250; 2,500; and 2,750 samples (when available in the training set), plus the dimension of the training set itself, which was used as the maximum dimension. When the size of the subsample was smaller than that of the training set, some samples were excluded from the training set and formed the test set. For each tissue, each clock was computed by considering males and females either jointly or separately. Finally, the clock trained on all blood samples was also evaluated on samples from other tissues to verify the possible advantage of tissue-specific clocks.

### 2.4 Evaluation of model performance

In order to evaluate the performance of the trained clock, we fitted a linear regression model of the computed methylage as a function of chronological age:

$$Methylage=\theta_{1}+\theta_{2}\cdot Age+residuals,$$ (1)

where *θ*_1_ and *θ*_2_ are the intercept and slope of the model, respectively. A visualization of the model is shown in Supplementary Figure S1.

Then, we evaluated the goodness of fit of such a model by computing two assessment measurements (generally called *Score* in the following sections): the root mean-squared error (RMSE) and the adjusted-*R* ^2^.

Given *n* observations *y*_*i*_ (*Methylage*) and given the predicted values $\hat{y_{i}}$ obtained from the model in Eq. 1 (predicted *Methylage*), the RMSE is defined as in Eq. 2

$$RMSE=\sqrt{\frac{1}{n}\overset{n}{\underset{i=1}{\sum }}{\left(y_{i}-\hat{y_{i}}\right)}^{2}}.$$ (2)

A graphical representation of the meaning of *y*_*i*_ (*Methylage*) and ${\hat{y}}_{i}$ (predicted *Methylage*) is shown in Supplementary Figure S1.

The RMSE, hence, describes how far apart the observed values are from the regression line (predicted values), on average. The lower the RMSE, the better the model fits the data, meaning that it is possible to accurately predict *Methylage* based on *Age*.

The adjusted-*R* ^2^ in Eq. 3 is a modified version of the *R* ^2^ that takes into account the number of predictors in the model (*K* = 2). It is computed as

$$Adjusted-R^{2}=1-\frac{\frac{1}{n-K}\sum_{i=1}^{n}{\left(y_{i}-\hat{y_{i}}\right)}^{2}}{\frac{1}{n-1}\sum_{i=1}^{n}{\left(y_{i}-\bar{y}\right)}^{2}},$$ (3)

where $\bar{y}=\frac{1}{n}\sum_{i=1}^{n}y_{i}$ . The adjusted-*R* ^2^ quantifies how well the predictor (*Age*) describes the dependent variable (*Methylage*). Its value ranges from 0 to 1, and it is related to the strength of the linear relationship between the two variables. A high adjusted-*R* ^2^, hence, indicates that the model fits the data well, meaning that *Methylage* and *Age* are highly correlated.

### 2.5 Comparison of model performance

In order to compare the performance of clocks computed under different scenarios, we fitted a series of mixed-effect models described in the following sections. The fitting was performed using the *lmer* function of the *lme*4 library v1.1.31 in R. In each model, a random intercept was introduced to take into account the dataset of origin of the samples (*GSE*). This is indicated as (1|*GSE*) in the formula of the models. Moreover, in all models, both the dependent and independent variables were always standardized, ensuring the interpretation of the partial slopes as partial correlations. Finally, in all models, we used the logarithm of the sample size instead of the sample size itself due to the skewness of its distribution.

#### 2.5.1 Dependence of the clock performance on the number of samples in the training set

To verify whether the predictive accuracy (*Score*) of the model evaluated on the validation set depends on the number of samples in the training set (*Sample size*), we considered all clocks trained and validated on males and females jointly and separately for each tissue and each penalty. In detail, we computed the mixed-effects model in Eq. 4 as follows:

$$Score∼log\left(Sample\;size\right)+\left(1|GSE\right).$$ (4)

The partial slope relative to *log*(*Sample size*) was then evaluated to assess the dependence of the *Score* on the sample size.

#### 2.5.2 Dependence of the clock performance on the type of penalization

To evaluate which among the ridge, lasso, and elastic-net penalization allowed us to obtain the clock with better predictive performance, we considered all the clocks trained and validated on males and females jointly, and we evaluated the relationship between the *Score* and the *Penalty* type by fitting for each tissue a -effects model as in Eq. 5

$$Score∼log\left(Sample\;size\right)+Penalty+\left(1|GSE\right),$$ (5)

where *log*(*Sample size*) is added to adjust the model for the number of samples in the training set. For each pair of penalties, a dummy variable *Penalty* is created so that its partial slope indicates how much the *Score* changes when choosing one penalty or the other.

#### 2.5.3 Comparison between sex-specific and universal clocks

To evaluate the possible advantage of a sex-specific clock, we considered for each tissue the results (*Score*) obtained training the elastic-net model on males and females jointly or separately. In both cases, the model was validated separately on males and females. Specifically, for each tissue, we fitted a mixed-effects model as follows in Eq. 6:

$$Score∼log\left(Sample\;size\right)+Sex.specific+\left(1|GSE\right),$$ (6)

where *Sex*.*specific* indicates whether the clock was sex-specific (1) or trained on males and females jointly (0) and *log*(*Sample size*) is added to adjust the model for the number of samples in the training set.

#### 2.5.4 Dependence of the clock performance on the tissue

In order to compare the predictive performance obtained in different tissues, for each pair of tissues, we fitted a mixed-effects model as follows in Eq. 7:

$$Score∼log\left(Sample\;size\right)+Tissue+\left(1|GSE\right),$$ (7)

where *Tissue* is a dummy variable appropriately defined for each pair of tissues and *log*(*Sample size*) is added to adjust the model for the number of samples in the training set. Here, we considered the *Score* obtained when keeping males and females jointly in the training and validation sets. Moreover, elastic-net-only results are presented owing to the better performance obtained, as per *Results* described below.

#### 2.5.5 Performance of the tissue-specific versus blood-trained clock

In order to compare the predictive performance of a tissue-specific clock and a clock trained on blood samples, for each tissue (except blood and whole blood, see “Datasets and tissues summary” in *Results* for their definition), we fitted a model as in Eq. 8

$$Score∼log\left(Sample\;size\right)+Tissue.specific+\left(1|GSE\right),$$ (8)

where *Tissue*.*specific* is a dummy variable equal to 0 if the model was trained on blood samples and to 1 if it was trained on the specific tissue and *log*(*Sample size*) is added to adjust the model for the number of samples in the training set. Here, we considered clocks trained on males and females jointly. Moreover, elastic-net-only results are presented owing to the better performance obtained, as per *Results* described below.

In all models, one-sample Student’s t-test was used to test the null hypothesis that the model coefficients are equal to zero. *p*-values were then adjusted using the Benjamini–Yekutieli (BY) approach (Benjamini and Yekutieli, 2001).

### 2.6 Comparison with other existing clocks

Methylage estimated by previously existing clocks was obtained using the EstImage webserver (Di Lena et al., 2021). These include Horvath13 (Horvath, 2013); PhenoAge (Levine et al., 2018); Zhang19.enpred and Zhang19.blupred (Zhang et al., 2019); Horvath18 (Horvath et al., 2018); Weidner14 (Weidner et al., 2014); Hannum13 (Hannum et al., 2013); ABEC, eABEC, and cABEC (Lee et al., 2020); Vidal (Vidal-Bralo et al., 2016); CorticalClock (Shireby et al., 2020); and MEAT (Voisin et al., 2020). A detailed description of all such clocks is given by Di Lena et al. (2022). The performance of the tissue-specific elastic-net clocks trained on males and females jointly (i.e., using the best performing setting according to our results) was then graphically compared with those of previously existing clocks using box plots.

## 3 Results

### 3.1 Datasets and tissues summary

As detailed in Supplementary Table S1, we found DNA methylation data of healthy samples passing our filters for four tissue categories: digestive system (A03), nervous system (A08), blood (A12.207.152), and adipose tissue (A10.165.114). However, only six samples (four females and two males) were available for the adipose tissue. Hence, this tissue was not included in the analysis. Table 1 summarizes the number of samples (females and males) found for each specific tissue.

**TABLE 1:** Summary of the number of samples found for each specific tissue for females (F) and males (M).

Tissue code	Tissue	F	M
A03.556.124.526.209.290	Supplementary Appendix	0	2
A03.556.124.526.356	Colon	0	1
A03.620	Liver	36	37
A08.186.211	Brain	3	3
A08.186.211.132.659	Mesencephalon	1	1
A08.186.211.132.810.428.200	Cerebellum	13	36
A08.186.211.200.885.287.249.487.550.184	Caudate nucleus	2	10
A08.186.211.200.885.287.500.270.548	Motor cortex	2	10
A08.186.211.200.885.287.500.382.500	Gyrus cinguli	2	10
A08.186.211.200.885.287.500.571	Occipital lobe	4	3
A08.186.211.200.885.287.500.571.735	Visual cortex	2	9
A08.186.211.200.885.287.500.670	Parietal lobe	4	19
A08.186.211.200.885.287.500.670.675	Somatosensory cortex	2	10
A08.186.211.200.885.287.500.863	Temporal lobe	5	10
A08.186.211.730.885.287.249.487.550.784	Putamen	10	32
A08.186.211.730.885.287.500.270	Frontal lobe	15	13
A08.186.211.730.885.287.500.270.700	Prefrontal cortex	127	250
A08.186.211.730.885.287.500.345	Hippocampus	6	12
A10.165.114	Adipose tissue	4	2
A12.207.152	Blood	1,389	1,209
A12.207.152.CD4	Blood CD4	30	7
A12.207.152.CD8	Blood CD8	28	3
A12.207.152.PBMC	Blood PBMC	19	4

Based on the number of samples per tissue, we focused our analysis on three tissues (liver, prefrontal cortex, and whole blood) and three aggregated tissues (digestive system, nervous system, and blood). In the second case, we also classified as blood samples those including only specific subsets of blood cells (CD4, CD8, or PBMC), and we included in the digestive system and nervous system all tissues that belong to such systems, according to the MeSH vocabulary (Lipscomb, 2000). Tables 2, 3 provide the number of samples available for each tissue.

**TABLE 2:** Summary of the number of samples for each tissue.

Tissue code	Tissue	F	M
A03.620	Liver	36	37
A08.186.211.730.885.287.500.270.700	Prefrontal cortex	122	235
A12.207.152	Whole blood	1,240	1,169

**TABLE 3:** Summary of the number of samples for each aggregated tissue. Here, blood also includes samples with only specific subsets of blood cells (CD4, CD8, or PBMC).

Tissue code	Tissue	F	M
A03	Digestive system	36	40
A08	Nervous system	162	335
A12.207.152	Blood	1,317	1,183

### 3.2 Improvement of the clock performance with the number of samples in the training set

**FIGURE 1:**
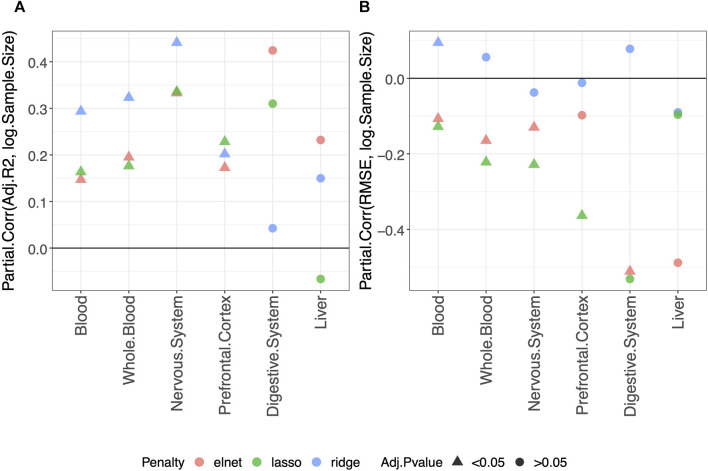
Model performance and sample size. Partial correlation (Partial.Corr) between the score of the model performance (adjusted-*R* ^2^ in **(A)** and RMSE in **(B)**), and the number of samples in the training set (log-transformed). As indicated in the legend, different colors refer to models trained with different penalties, while different symbols refer to one-sample *t*-test BY adjusted *p*-values (Adj.*p*-value) greater or smaller than the significance threshold 0.05 (see *Materials and methods* for details). As expected, the performance improves when the sample size is increased.

Using the approach described in *Materials and methods*, we statistically evaluated the dependency of the clock performance on the sample size (Supplementary Figures S2–S7 provide an overview of such dependency). The partial correlations between each *Score* and the logarithm of the sample size (*log*(*Sample size*)) are reported in Supplementary Table S2, which includes results obtained in the validation set and the training and test sets. The results for the adjusted-*R* ^2^ and RMSE obtained in the validation set are shown in Figure 1. Figure 1A shows that, overall, a positive correlation (Partial.Corr) exists between the model adjusted-*R* ^2^ on the validation set and the number of samples in the training set. The effect is evident for all three types of penalization, and it is statistically significant in blood, whole blood, the nervous system, and prefrontal cortex but not in the digestive system and liver. Figure 1B shows that, overall, the RMSE on the validation set decreases as the number of samples in the training set increases (negative Partial.Corr), confirming that the performance of the model on the validation set improves when the sample size of the training set increases. More precisely, such a trend is not visible when using ridge penalization, where the only statistically significant result is in blood and shows an opposite behavior, while the results obtained with the lasso and elastic-net are in agreement with the overall trend, although no statistical significance is found in the digestive system and liver when using the lasso and in the prefrontal cortex and liver when using the elastic-net penalization.

It should be noted that the non-significant effect of the sample size in the digestive system and liver is possibly due to the small number of samples available for such tissues (Tables 2, 3 show for the number of samples available for each tissue and aggregated tissue, and Supplementary Table S1 shows the number of studies for each tissue and the number of samples available within each study).

### 3.3 Using the elastic-net rather than other penalizations does not affect the clock performance

We evaluated the impact of using different penalties on the predictive performance of the clock computing the partial correlation between the *Score* and the *Penalty* of the clocks, as described in *Materials and methods*. Specifically, for each pair of penalties, we obtained an estimate of the partial correlation (Partial.Corr) describing how much the *Score* changes when changing the *Penalty*.

We considered the *Score* values obtained on the validation set. However, results on the training and test sets are also available and given in Supplementary Table S3.

Average values of adjusted-*R* ^2^ and RMSE per penalty within each tissue are given in Supplementary Tables S4, S5.

**FIGURE 2:**
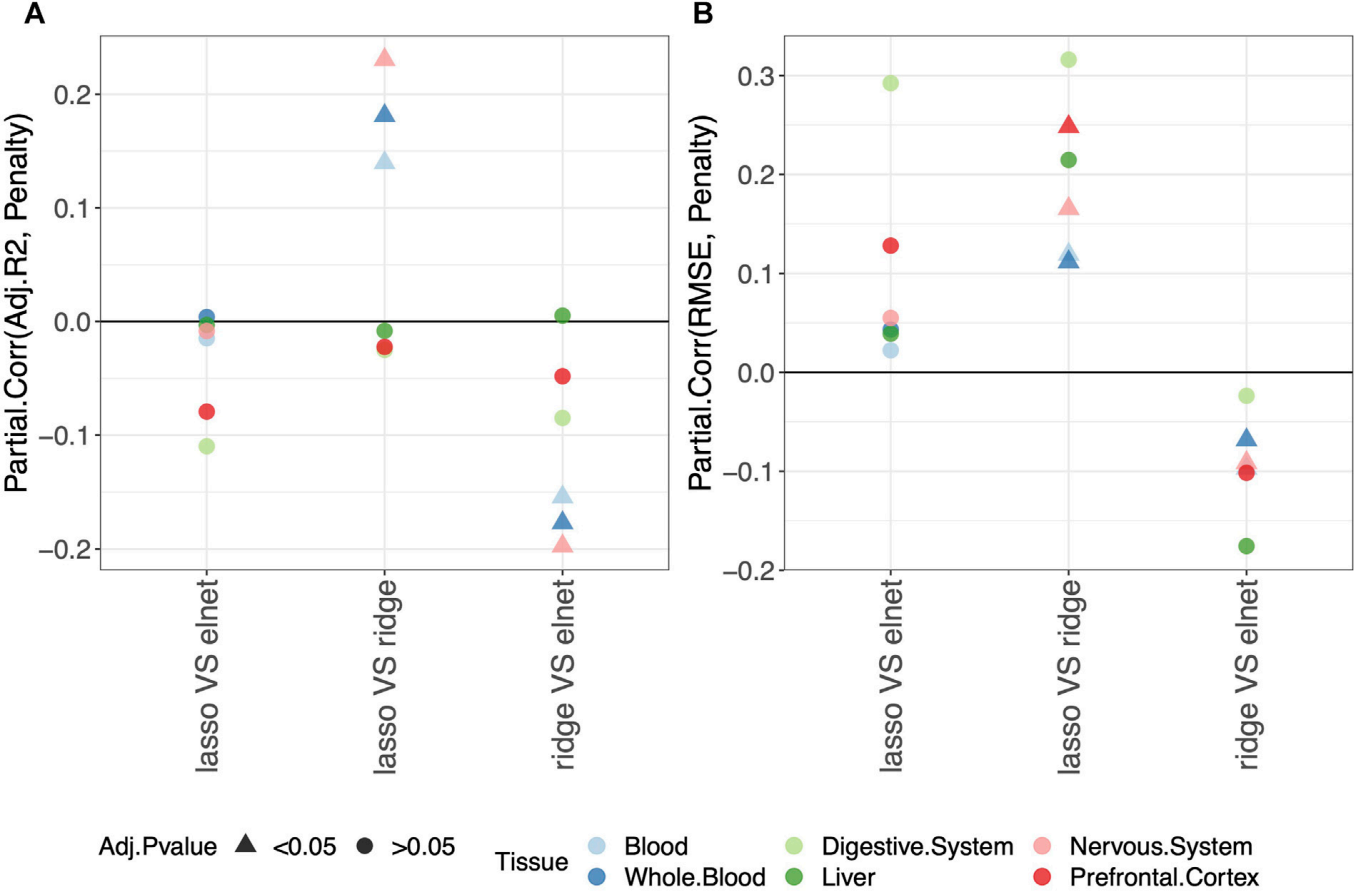
Model performance and penalty. Partial correlation between score (adjusted-*R* ^2^ in **(A)** and RMSE in **(B)**) and penalty. The model was fitted (and validated) considering males and females jointly. The dummy variables in the model are labeled as follows: “ridge V elnet” means ridge = 1, elnet = 0; “lasso VS elnet” means lasso = 1, elnet = 0; and “lasso VS ridge” means lasso = 1, ridge = 0. As indicated in the legend, different colors refer to different tissues, while different symbols refer to one-sample *t*-test BY adjusted *p*-values (Adj.*p*-value) greater or smaller than the significance threshold 0.05 (see *Materials and methods* for details). Overall, choosing the elastic-net penalization does not affect the clock performance.

Regarding the comparison between the lasso and elastic-net penalization, Figure 2 shows that both adjusted-*R* ^2^ and RMSE improve when using the elastic-net, but the differences are not statistically significant. Choosing the ridge penalization over the lasso or the elastic-net, on the other hand, reduces the RMSE while also reducing the adjusted-*R* ^2^. This implies that the correlation between methylage and real age is lower when using the ridge penalization, even though the uncertainty of such correlations is also reduced.

It should be noted that when considering the digestive system or the liver, all *p*-values are >0.05 , possibly due to the small sample size. However, the trend of the results is in agreement with those obtained in the other tissues.

Overall, we conclude that the common choice of using the elastic-net penalization does not impact the results when aiming to obtain a predictive model of methylage that is in agreement with the real age. Hence, in the following sections, we focus on this penalization. The results for the other penalizations are reported in Supplementary Tables, as detailed in the following sections.

### 3.4 Sex-specific clocks do not perform better than clocks trained on males and females jointly

For each tissue, we evaluated the possible advantage of a sex-specific clock in predicting methylage in the validation set, using the approach described in *Materials and methods*. The results comparing the clocks trained on males and females jointly or separately for males and females are shown in Supplementary Table S6. Average values of adjusted-*R* ^2^ and RMSE per tissue and per sex (i.e., sex selected in the training and validation sets) are shown in Supplementary Tables S7, S8.

**FIGURE 3:**
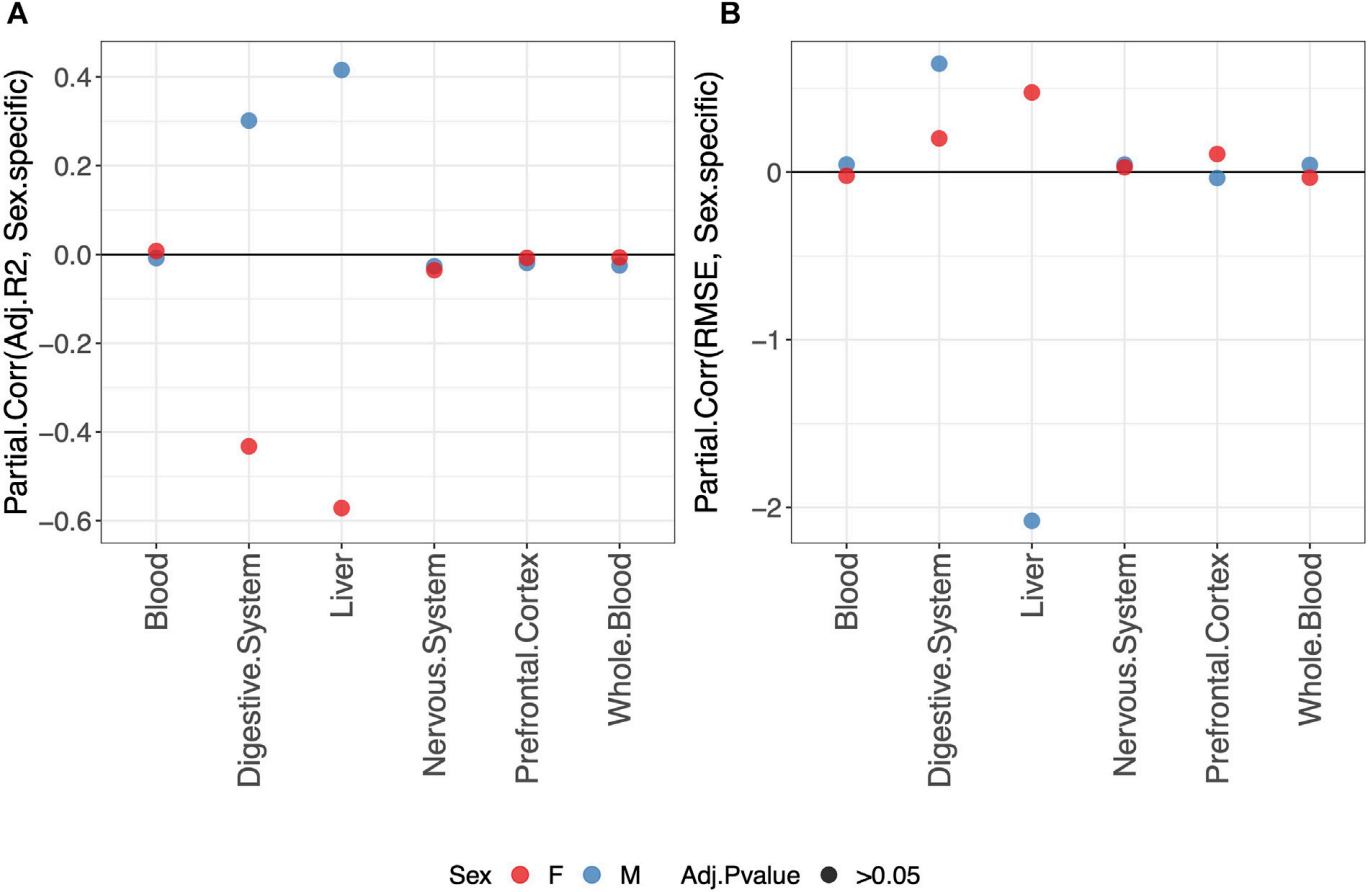
Partial correlation between the score (adjusted-*R* ^2^ in **(A)** and RMSE in **(B)** and model either fitted jointly on males and females (0) or separately for males (blue) or females (red) (1). The model was trained using the elastic-net penalization. The results are shown for the validation set. A positive partial correlation for the adjusted-*R* ^2^ means that the performance is higher when fitting a sex-specific clock. The opposite holds for RMSE. In all cases, the one-sample *t*-test BY adjusted *p*-value (Adj.*p*-value) is >0.05 , and the null hypothesis that the partial correlation is zero cannot be rejected.

Overall, we do not detect any statistically significant difference in the predictive performance of clocks when fitting a sex-specific clock or a clock for males and females jointly (Figure 3).

### 3.5 Difference between results obtained in different tissues

We compared the predictive performance on the validation set of each pair of tissues, as described in *Materials and methods*. The results are shown in Supplementary Table S9. The average values of adjusted-*R* ^2^ and RMSE per tissue are shown in Supplementary Tables S10, S11.

**FIGURE 4:**
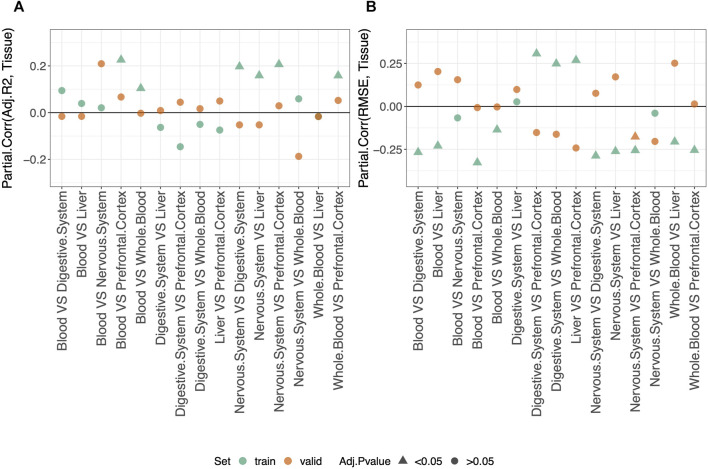
Partial correlation between the score (adjusted-*R* ^2^ in **(A)** and RMSE in **(B)** and having fitted/validated the model on one tissue or the other. As indicated in the legend, different colors refer to results obtained for the training or validation set, while different symbols refer to one-sample *t*-test BY adjusted *p*-values (Adj.*p*-value) greater or smaller than the significance threshold 0.05 (see *Materials and methods* for details). The model was trained on males and females jointly using the elastic-net penalization. Given the label “tissue1 VS tissue2,” a negative Partial.Corr for the adjusted-*R* ^2^ means that the performance is lower in the first tissue (tissue1), while the opposite holds for the RMSE.

Considering the results on the validation set (orange dots in Figure 4), we do not detect any difference in the performance of different tissue-specific clocks.

### 3.6 Training a tissue-specific model improves the clock performance

Considering the easier clinical translatability of a clock computed on blood compared to other tissues, we evaluated whether methylation information from blood samples is already effective in making blood-specific clocks comparable to their tissue-specific counterparts. To this aim, for each tissue (except blood and whole blood), we compared the predictive performance on such tissues of a model trained on blood or on the specific tissue. The details are provided in *Materials and methods*. The results comparing the clocks trained on blood or on specific tissues are shown in Supplementary Table S12. Average values of adjusted-*R* ^2^ and RMSE per tissue are shown in Supplementary Tables S13, S14.

**FIGURE 5:**
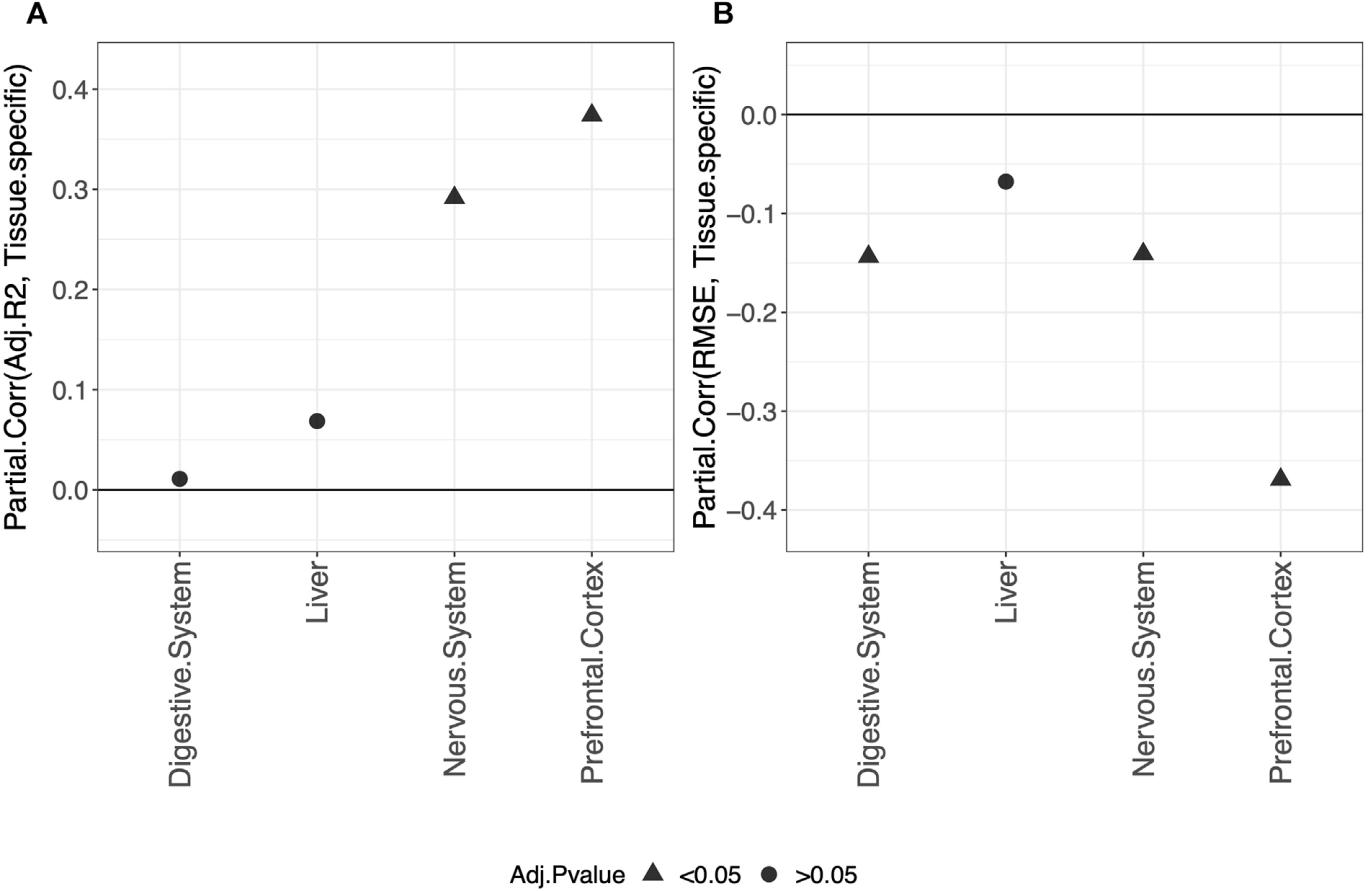
Partial correlation between the score (adjusted-*R* ^2^ in **(A)** and RMSE in **(B)** and having trained the model on the specific tissue (1) or on blood (0). As indicated in the legend, different symbols refer to one-sample *t*-test BY adjusted *p*-values (Adj.*p*-value) greater or smaller than the significance threshold 0.05 (see *Materials and methods* for details). The model was trained on males and females jointly using elastic-net penalization. The performance was evaluated on the validation set (tissue indicated in the x-axis). A positive partial correlation for adjusted-*R* ^2^ means that the performance is higher when fitting a tissue-specific clock, while the opposite holds for the RMSE.

Overall, our results show that tissue-specific clocks perform better than the blood-specific clock. Figure 5 shows, in fact, that fitting a tissue-specific clock results in an increase in the adjusted-*R* ^2^ and a decrease in the RMSE. The results for the digestive system and liver are not statistically significant due to the small sample size but have the same trend observed for the nervous system and prefrontal cortex.

### 3.7 Comparison with other clocks

Based on the previous results, we selected the elastic-net tissue-specific clocks trained on males and females jointly (this setting was chosen because of their better performance, knowing that joint male and female individuals increase the sample size) and without performing any subsampling, and we compared the performance of such clocks with that of previously existing clocks by evaluating the concordance between methylage and real age on the validation sets using the adjusted-*R* ^2^ and RMSE. Supplementary Figures S8, S13 show the results for each tissue. Overall, the performance on the validation set has high variability for most clocks. The graphical comparison provided by the box plots highlights the utility of tissue-specific clocks. While the elastic-net clocks trained on the liver and digestive system provide worse predictions than multi-tissue and blood-specific clocks (Supplementary Figures S8, S9), possibly due to the small sample size of the training set, the tissue-specific elastic-net models for the nervous system and prefrontal cortex achieve particularly good performance, together with the CorticalClock, a clock specific for the cerebral cortex (Supplementary Figures S10, S11). Regarding the blood-specific elastic-net clock, Supplementary Figures S12, S13 show that the results obtained using the elastic-net model are comparable with those obtained using most of the other blood-specific clocks (except for Weidner14 and Vidal, two clocks based on the methylation value of only a few CpGs: three in the case of Weidner14 and eight in the case of Vidal), as well as with multi-tissue clocks.

### 3.8 CpGs and corresponding genes selected by the tissue-specific clocks

Considering, as in the previous section, the tissue-specific elastic-net models trained on males and females jointly and without performing any subsampling, we retrieved the list of CpGs selected by each clock for the computation of methylage. Overall, we observe that the number of selected CpGs increases with the sample size of the training set. This is an expected result related to an increase in the test power. The selected CpGs and their partial effects within each clock are shown in Supplementary Figures S14–S19. Here, all coefficients between −0.1 and 0.1 were set to 0 to simplify the heatmaps. Moreover, the less stable CpGs were discarded by removing the CpGs with non-zero coefficients in less than 10% of the clocks within a certain tissue.

**FIGURE 6:**
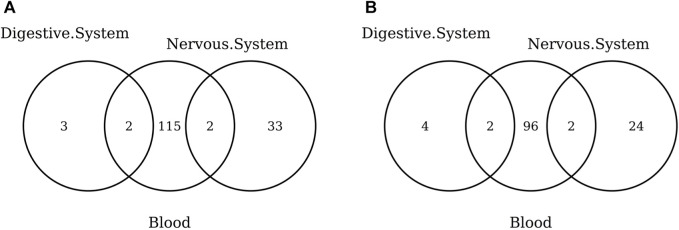
Venn diagram of CpGs **(A)** and genes **(B)** shared in blood, the nervous system, and digestive system. Here, we considered the results obtained using the elastic-net tissue-specific clocks trained on males and females jointly and without performing any subsampling. The Venn diagrams were then obtained taking into account only the CpGs selected in all the training sets for each tissue.

**FIGURE 7:**
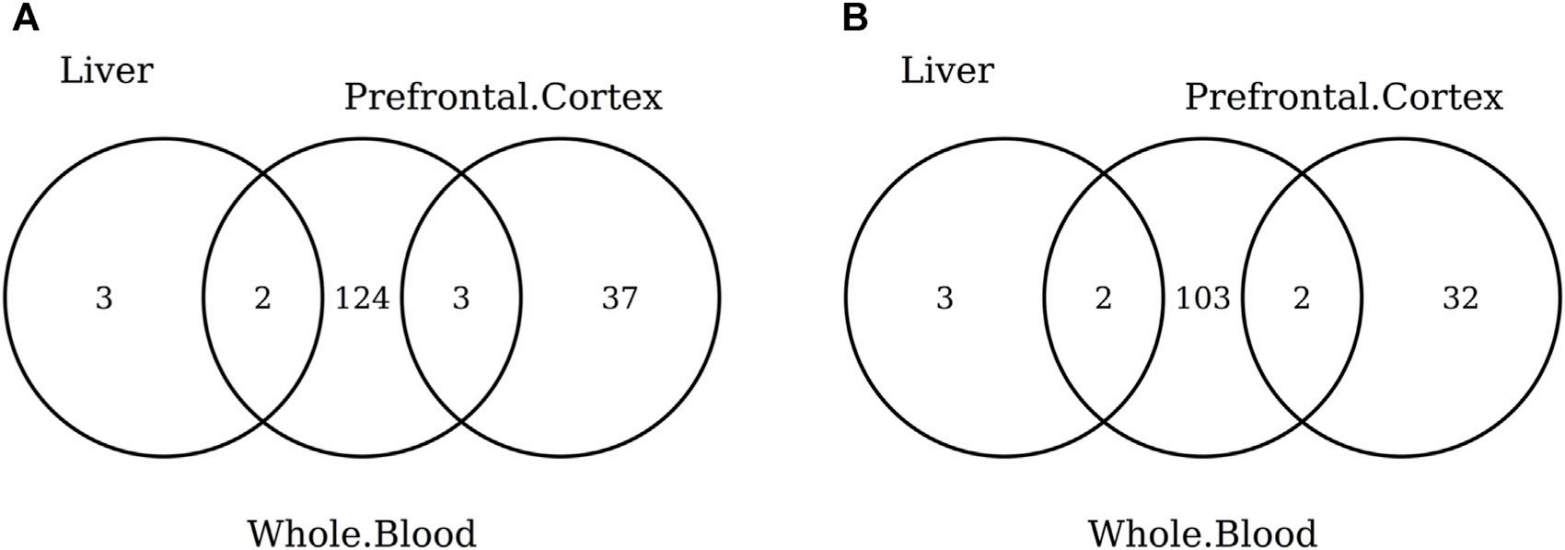
Venn diagram of CpGs **(A)** and genes **(B)** shared in whole blood, the prefrontal cortex, and liver. Here, we considered the results obtained using the elastic-net tissue-specific clocks trained on males and females jointly and without performing any subsampling. The Venn diagrams were then obtained taking into account only the CpGs selected in all the training sets for each tissue.

The list of the CpGs selected by all elastic-net clocks for each separate tissue is given in Supplementary Table S15. Overall, no CpG is selected by all elastic-net clocks in all tissues. The Venn diagram given in Figure 6A shows that, while a couple of CpGs are selected both in blood and the digestive system or nervous system, none of them are shared between the last two tissues. A very similar result is obtained when grouping the CpGs at the gene level (Figure 6B) or when considering more specific tissues (Figure 7). Our results confirm the high heterogeneity of the selected CpGs and genes. An overview of the genes selected by both previously existing clocks and those found in our elastic-net tissue-specific models is provided in Supplementary Table S15.

**FIGURE 8:**
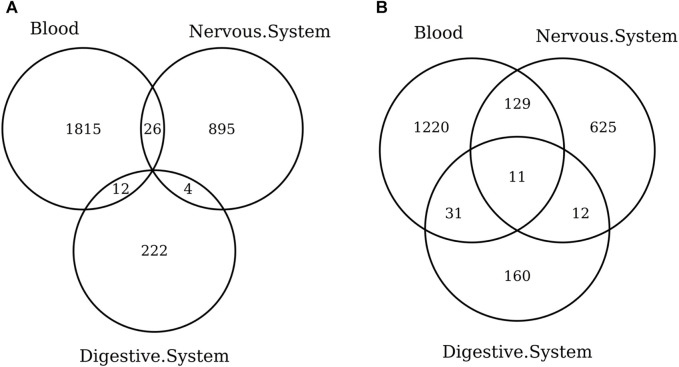
Venn diagram of CpGs **(A)** and genes **(B)** shared in blood, the nervous system, and digestive system. Here, we considered the results obtained using the elastic-net tissue-specific clocks trained on males and females jointly and without performing any subsampling. The Venn diagrams were then obtained taking into account all the CpGs selected in at least one of the training sets for each tissue.

**FIGURE 9:**
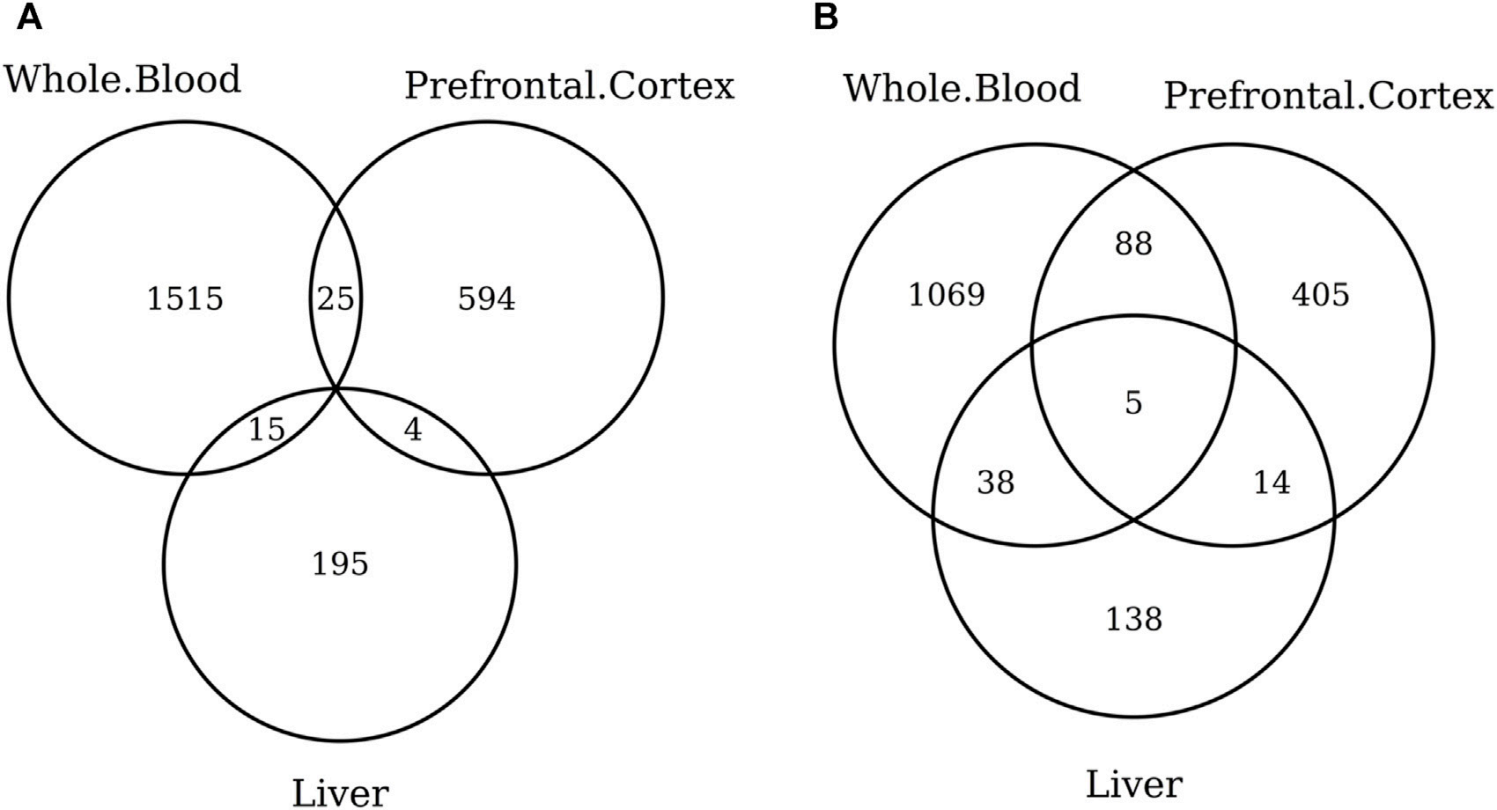
Venn diagram of CpGs **(A)** and genes **(B)** shared in whole blood, the prefrontal cortex, and liver. We considered the results obtained using the elastic-net tissue-specific clocks trained on males and females jointly and without performing any subsampling. The Venn diagrams were then obtained taking into account all the CpGs selected in at least one of the training sets for each tissue.

When considering the CpGs selected in at least one elastic-net clock, we find some CpGs shared by each pair of tissues but no CpG shared by all the three tissues (Figures 8A, 9A). Figures 8B, 9B show instead that, when aggregating the CpGs at the gene level, we identify 11 genes that are selected in all the three aggregated tissues (CPT1A, EIF5A2, ELOVL2, FHL2, FLJ45983, GATA3, MMP15, SHROOM3, SLC9A3, SLIT3, and SYNGR3) and 5 genes that are selected in all the 3 specific tissues (CPLX2, HAS3, MMP15, RPA2, and SYNGR3). Interestingly, ELOVL2 and FHL2 are well-known as epigenomic markers of aging (Garagnani et al., 2012) and are also selected by various other existing methylage clocks (Table 4). Furthermore, Table 4 shows that among the remaining 12 genes, 7 are also selected by at least 1 other methylage clock, while 5 are uniquely identified by the elastic-net clocks (CPT1A, MMP15, SHROOM3, SLC9A3, and SLIT3).

**TABLE 4:** Column “Gene” lists the 14 genes found in at least 1 clock in all 3 tissues or aggregated tissues. For each gene, column “Clock” lists the previously existing clocks in which such a gene was also used for the computation of methylage.

Gene	Clock
CPT1A	
EIF5A2	cABEC
ELOVL2	Zhang19.enpr; Horvath18; Hannum13; eABEC; cABEC; ABEC; CorticalClock; and ELOVL2
FHL2	Zhang19.enpr; Horvath18; Hannum13; eABEC; cABEC; ABEC; and FHL2
FLJ45983	CorticalClock
GATA3	CorticalClock
MMP15	
SHROOM3	
SLC9A3	
SLIT3	
SYNGR3	Zhang19.enpr and eABEC
CPLX2	ABEC
HAS3	PhenoAge
RPA2	Zhang19.enpr; Horvath18; Hannum13; eABEC; cABEC; and ABEC

Zhang19.blupred was not considered in this table due to the high number of selected CpGs ( > 300k). The complete inventory of genes selected by each methylage clock is given in Supplementary Table S15, together with the list of clocks using each respective gene.

## 4 Discussion

Methylage clocks are novel biomarkers of aging (He et al., 2021), which exploit DNA methylation to estimate biological age. A common approach to develop such clocks is to fit a penalized linear regression model that relates the chronological age of individuals with their DNA methylation values, considering a training set of control samples. In addition to the relevance of methylage clocks, important aspects of their computational strategy are not well-defined. These include the criteria by which the training set samples are selected (e.g., males and females jointly or separately, samples from a specific tissue or from different tissues) and the model specifications (e.g., the type of penalty). Our work aims at providing a broad analysis of the dependency of methylage clock performance on a number of relevant variables and identifies different significant aspects. Among such aspects, the sample size of the training set is indeed a critical point; as expected, a larger sample size improves the predictive performance of the clock. For what regards the choice of the penalty type, no statistically significant difference is observed among the ridge, lasso, and elastic-net penalization, confirming the suitability of the most commonly used elastic-net approach. In addition to the well-known differences in the aging process in males and females (Yusipov et al., 2020; Hägg and Jylhävä, 2021; Iannuzzi et al., 2023), our results do not find a significant advantage in computing a sex-specific clock, meaning that methylage clocks capture a broader signal, which is sex-independent. Another kind of unexpected result is that the performance of diverse tissue-specific clocks is not different. Since the aging process is linked to multiple cellular alterations, which frequently exhibit specificity for particular tissues (Ferrucci et al., 2020; Nie et al., 2022), we might expect to obtain stronger signatures of aging in some tissues than in others. However, this is not the case for methylage clocks, according to our results. Interestingly, on the other hand, tissue-specific clocks perform better than a generic clock trained on blood. Blood is indeed a favored tissue for the computation of methylage clocks due to its ease of sampling. However, such differences in performance should be taken into account when generalizing the results obtained on blood to other tissues or the whole body. The importance of tissue specificity also emerges when comparing the results obtained with our clocks with those achieved with other existing clocks. For instance, despite the high variability in the predictive performance of the clocks, it emerges that the CorticalClock works better than clocks trained on other tissues when predicting the age of prefrontal cortex and nervous system samples.

Finally, our results show a high variability in the biological signal taken into account by different clocks. Considering the sets of selected CpGs, we find, for instance, that no CpG is shared by all tissue-specific clocks. However, when we shift at the gene level and compare the genes to which the selected CpGs are associated, we find some commonality: 5 genes are selected in all the clocks obtained from the 3 considered specific tissues (liver, prefrontal cortex, and whole blood), while 11 genes are selected in all the clocks obtained from the 3 considered aggregated tissues (digestive system, nervous system, and blood). Among those common genes, ELOVL2 and FHL2 stand out since they are two well-known markers of aging (Garagnani et al., 2012). Moreover, the majority of genes (9 out of 14) are also selected by other existing clocks. On the other hand, CPT1A, MMP15, SHROOM3, SLC9A3, and SLIT3 are first identified within our clocks. Although the identification of such genes can be considered by no means conclusive as to their power as methylage markers, they remain a robust set of hypotheses for further (experimental) testing.

To conclude, we mention that the limits of our study are clearly related to the usage of data obtained using a single technology (Illumina HumanMethylation450 BeadChip) and model (penalized linear regression), as well as to the data availability per tissue. Furthermore, additional experiments like assessing the performance of blood clocks using only CpGs linked to genes expressed in all tissues, or pre-selected according to other biological criteria (for instance, the remarkable case of genes associated with sex-specific hormones), or testing tissue-specific models on different tissues, represent as many questions that could provide additional insights into the mechanisms of methylage. Nevertheless, such a systematic analysis represents a useful and needed evidence-based ground for additional and further exploration in the translation of epigenetic clocks.

## Data Availability

Publicly available datasets were analyzed in this study. These data can be found at: Gene Expression Omnibus (GEO) repository (https://www.ncbi.nlm.nih.gov/geo/) under the IDs listed in Supplementary Table S1.
