# Supplementary material for: Where are we in the implementation of tissue-specific epigenetic clocks?
Source: Front Bioinform. 2024 Mar 4;4:1306244. doi: 10.3389/fbinf.2024.1306244 (PMC10944965; doi:10.3389/fbinf.2024.1306244)
Supplement: Supplementary file 7 [file DataSheet1.PDF]

Supplementary Material

1 SUPPLEMENTARY FIGURES

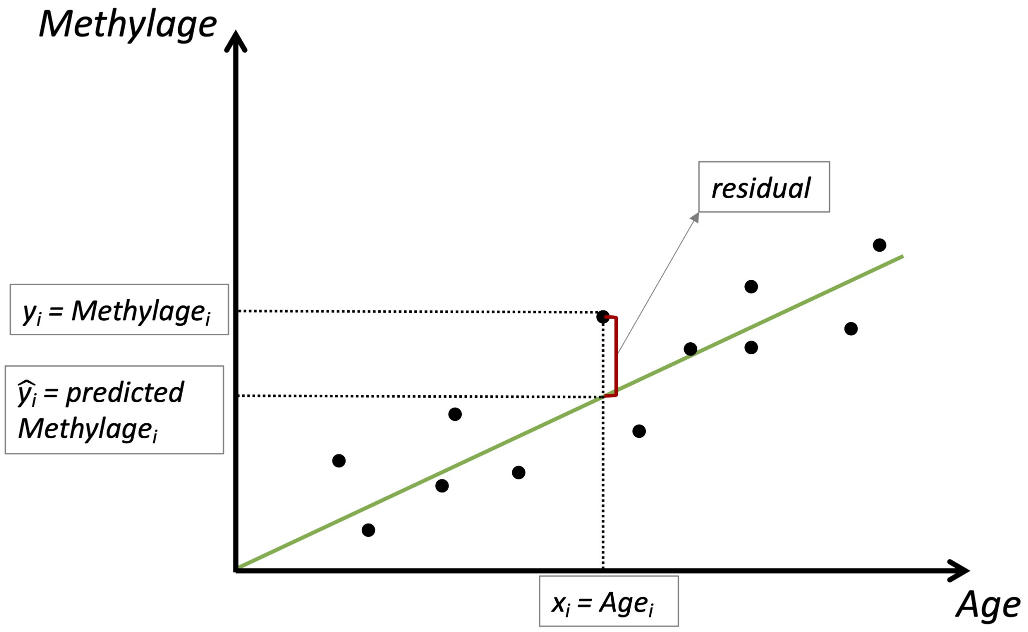

Figure S1. Example of linear regression between $Methylage$ and Age using fake data fabricated for visualization purposes. Each dot corresponds to a sample. For a specific sample (i), the values of $Methylage_i$ (in the equations referred to as y_i), Age_i, and the predicted $Methylage_i$ obtained from the regression line (in the equations referred to as $\hat{y}_i$) have been highlighted. The residual for sample i corresponds to the distance between its point and the regression line and it is highlighted in red.

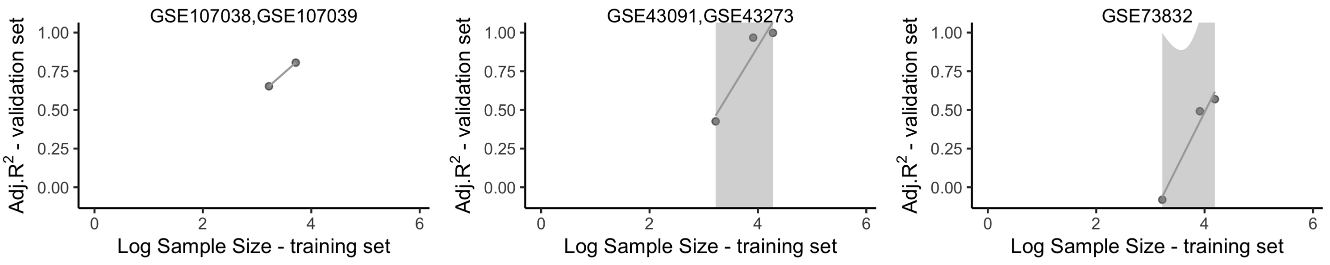

Figure S2. Plot showing the linear relation between the Adjusted R^2 obtained on the validation set and the logarithm of the number of samples in the training set in Digestive System. Here, we consider the model trained with the elastic-net penalization on males and females jointly. The name of the validation set is specified in the subplot title. Only validation sets with at least 3 samples were considered.

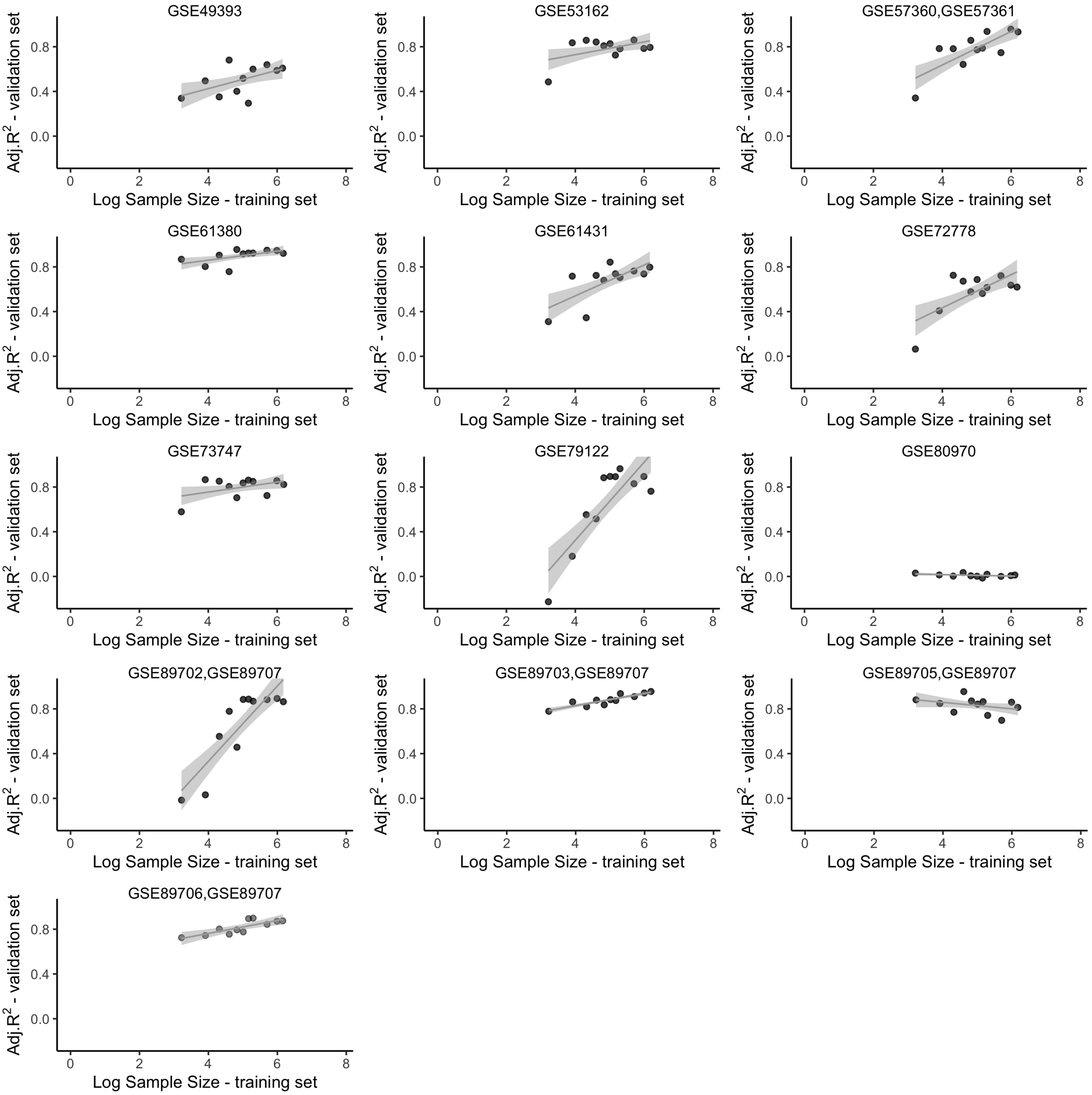

Figure S3. Plot showing the linear relation between the Adjusted R^2 obtained on the validation set and the logarithm of the number of samples in the training set in Nervous System. Here, we consider the model trained with the elastic-net penalization on males and females jointly. The name of the validation set is specified in the subplot title. Only validation sets with at least 3 samples were considered.

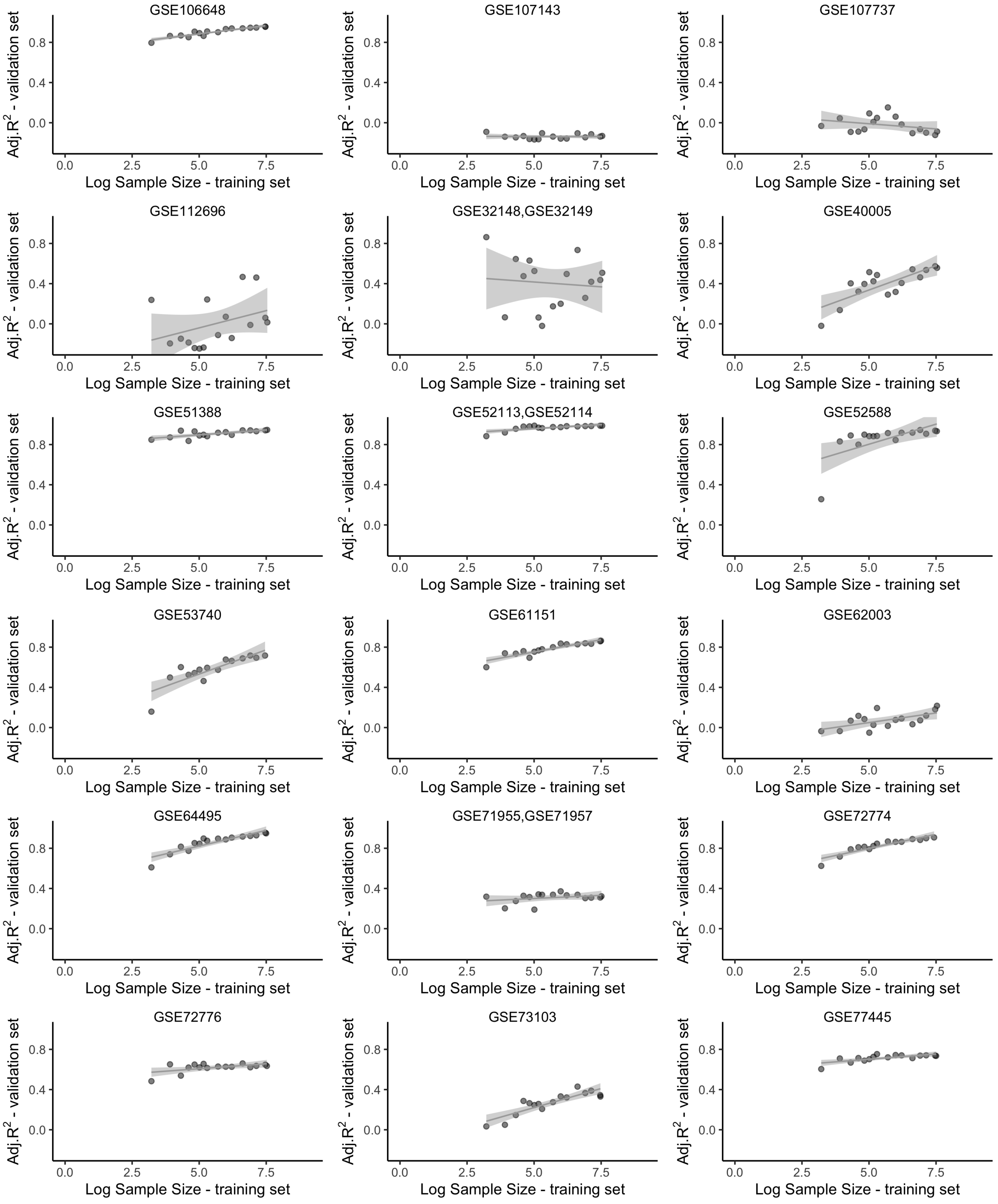
GSE106648
GSE107143
GSE107737
GSE112696
GSE32148,GSE32149
GSE40005
GSE51388
GSE52113,GSE52114
GSE52588
GSE53740
GSE61151
GSE62003
GSE64495
GSE71955,GSE71957
GSE72774
GSE72776
GSE73103
GSE77445
Adj.R^2 - validation set
Log Sample Size - training set

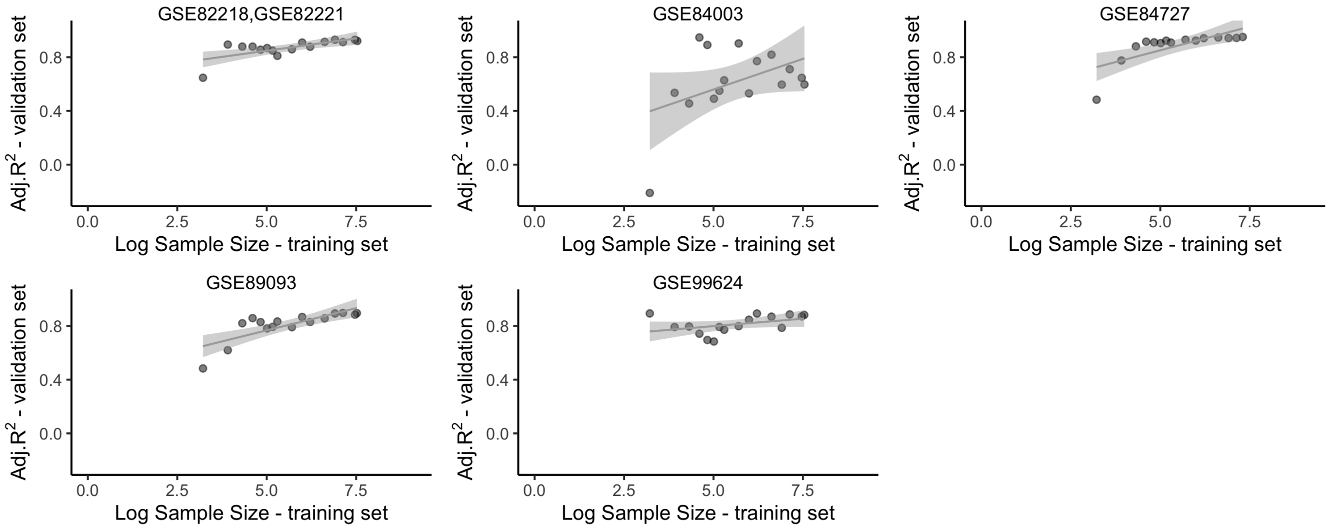

Figure S4. Plot showing the linear relation between the Adjusted R^2 obtained on the validation set and the logarithm of the number of samples in the training set in Blood. Here, we consider the model trained with the elastic-net penalization on males and females jointly. The name of the validation set is specified in the subplot title. Only validation sets with at least 3 samples were considered.

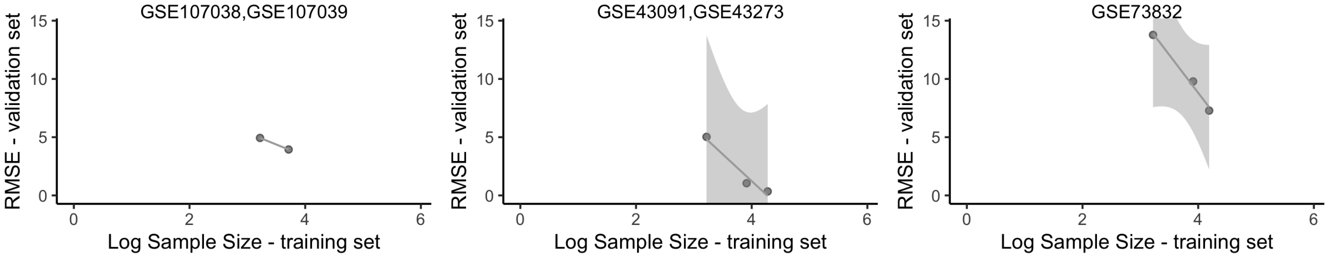

Figure S5. Plot showing the linear relation between the RMSE obtained on the validation set and the logarithm of the number of samples in the training set in Digestive System. Here, we consider the model trained with the elastic-net penalization on males and females jointly. The name of the validation set is specified in the subplot title. Only validation sets with at least 3 samples were considered.

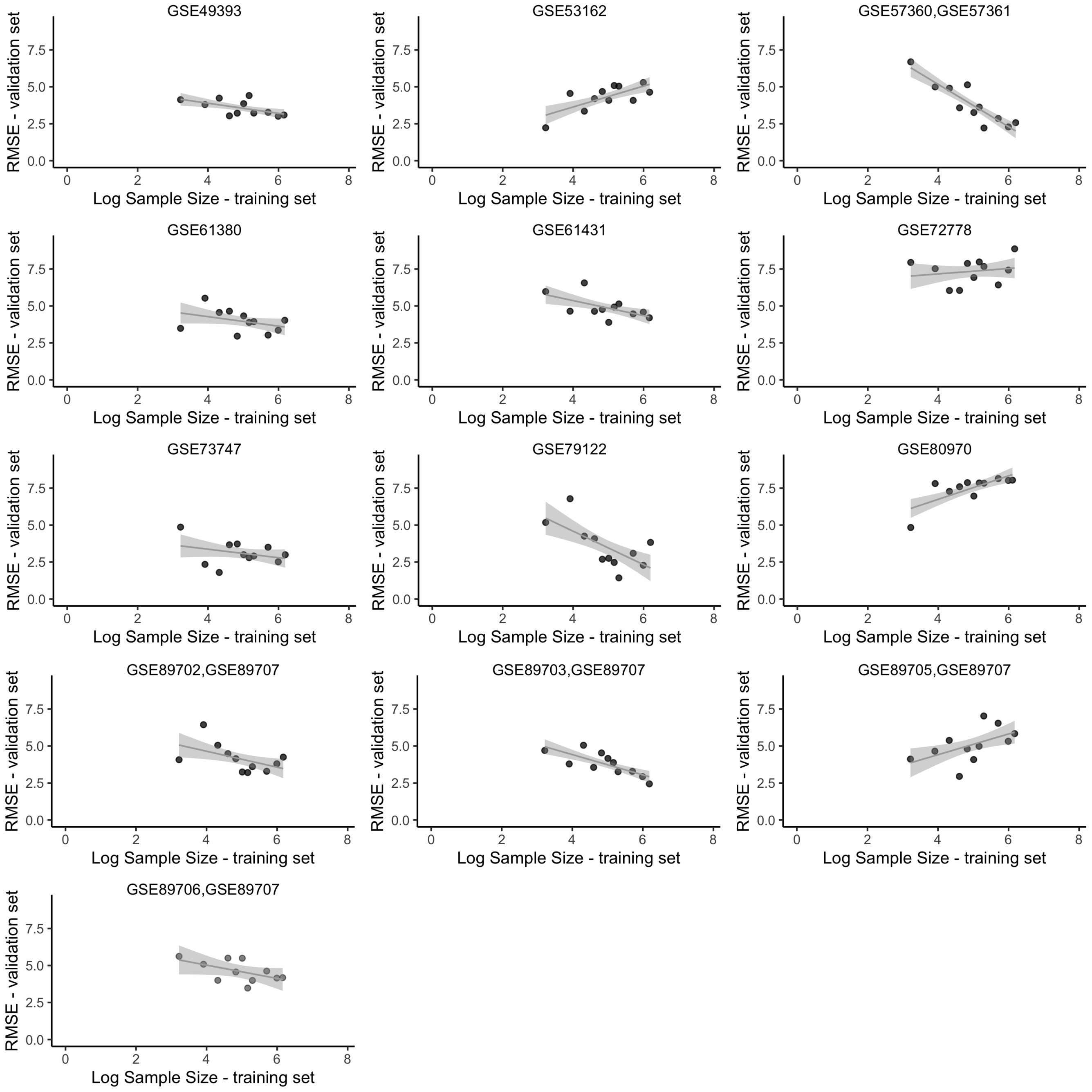

Figure S6. Plot showing the linear relation between the RMSE obtained on the validation set and the logarithm of the number of samples in the training set in Nervous System. Here, we consider the model trained with the elastic-net penalization on males and females jointly. The name of the validation set is specified in the subplot title. Only validation sets with at least 3 samples were considered.

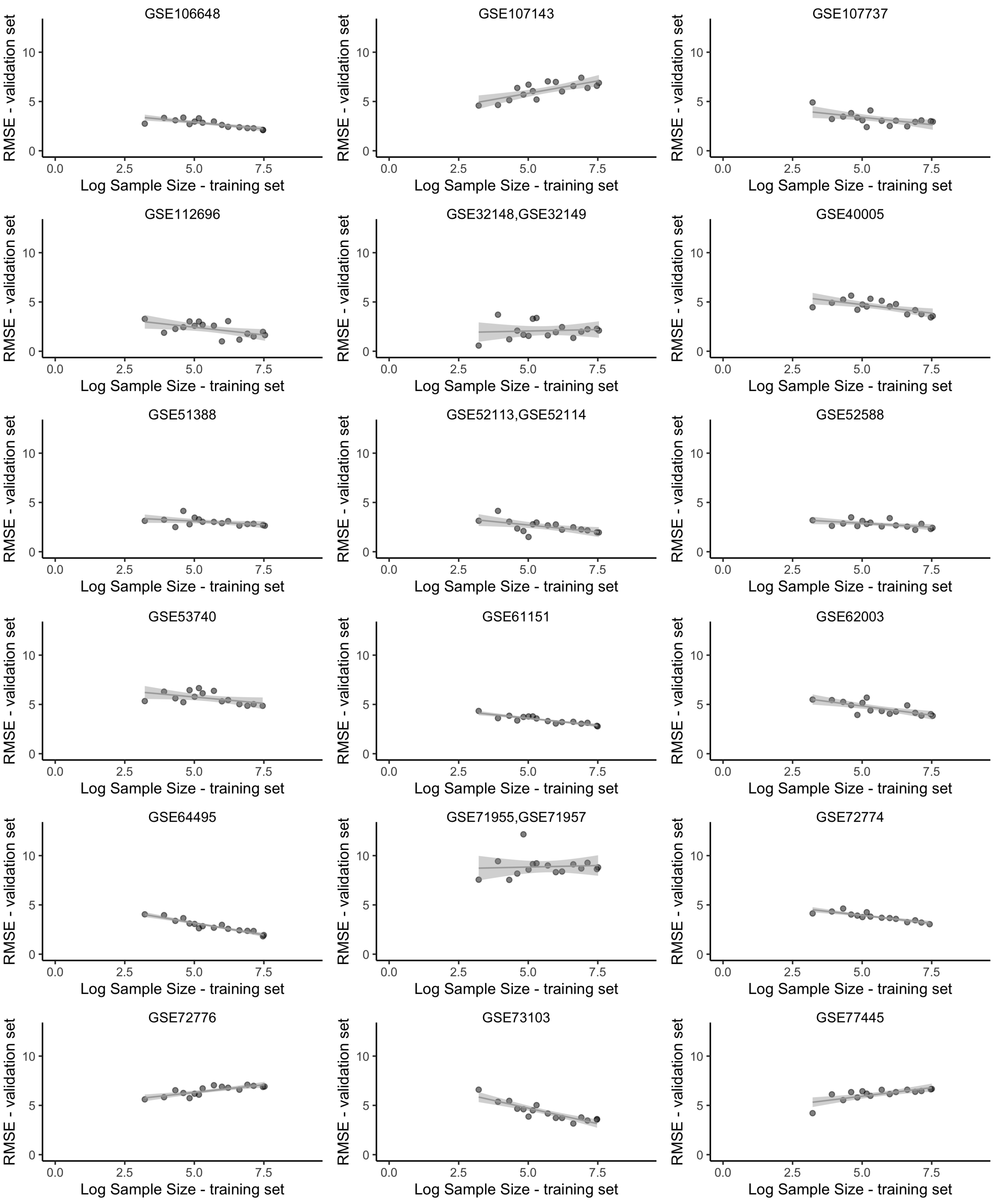
GSE106648
GSE107143
GSE107737
GSE112696
GSE32148,GSE32149
GSE40005
GSE51388
GSE52113,GSE52114
GSE52588
GSE53740
GSE61151
GSE62003
GSE64495
GSE71955,GSE71957
GSE72774
GSE72776
GSE73103
GSE77445
RMSE - validation set
Log Sample Size - training set
0
5
10
0.0
2.5
5.0
7.5

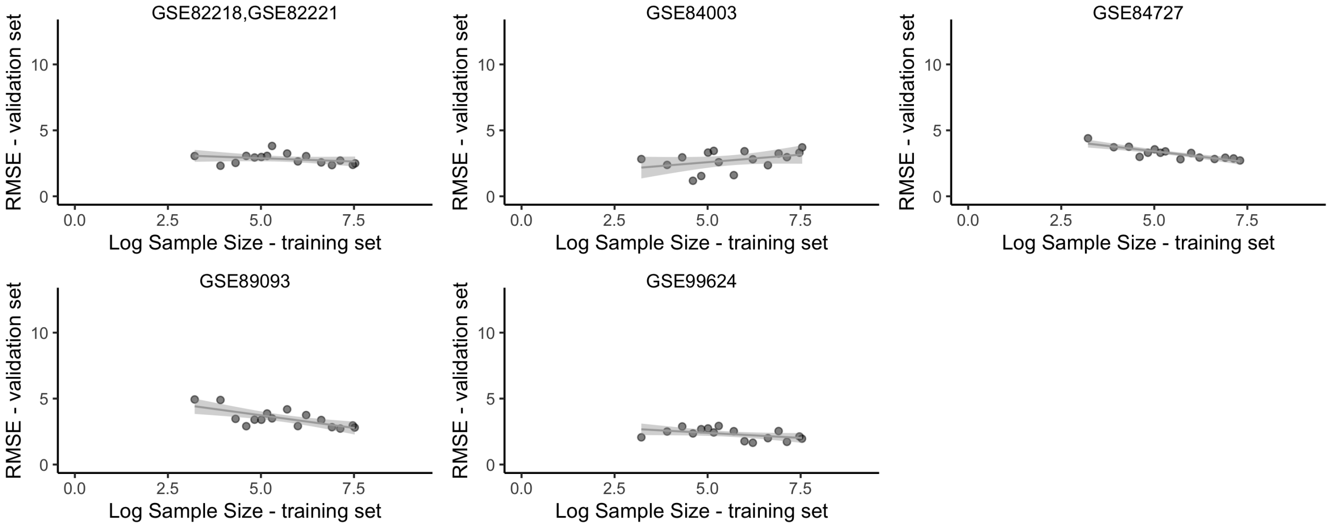

Figure S7. Plot showing the linear relation between the RMSE obtained on the validation set and the logarithm of the number of samples in the training set in Blood. Here, we consider the model trained with the elastic-net penalization on males and females jointly. The name of the validation set is specified in the subplot title. Only validation sets with at least 3 samples were considered.

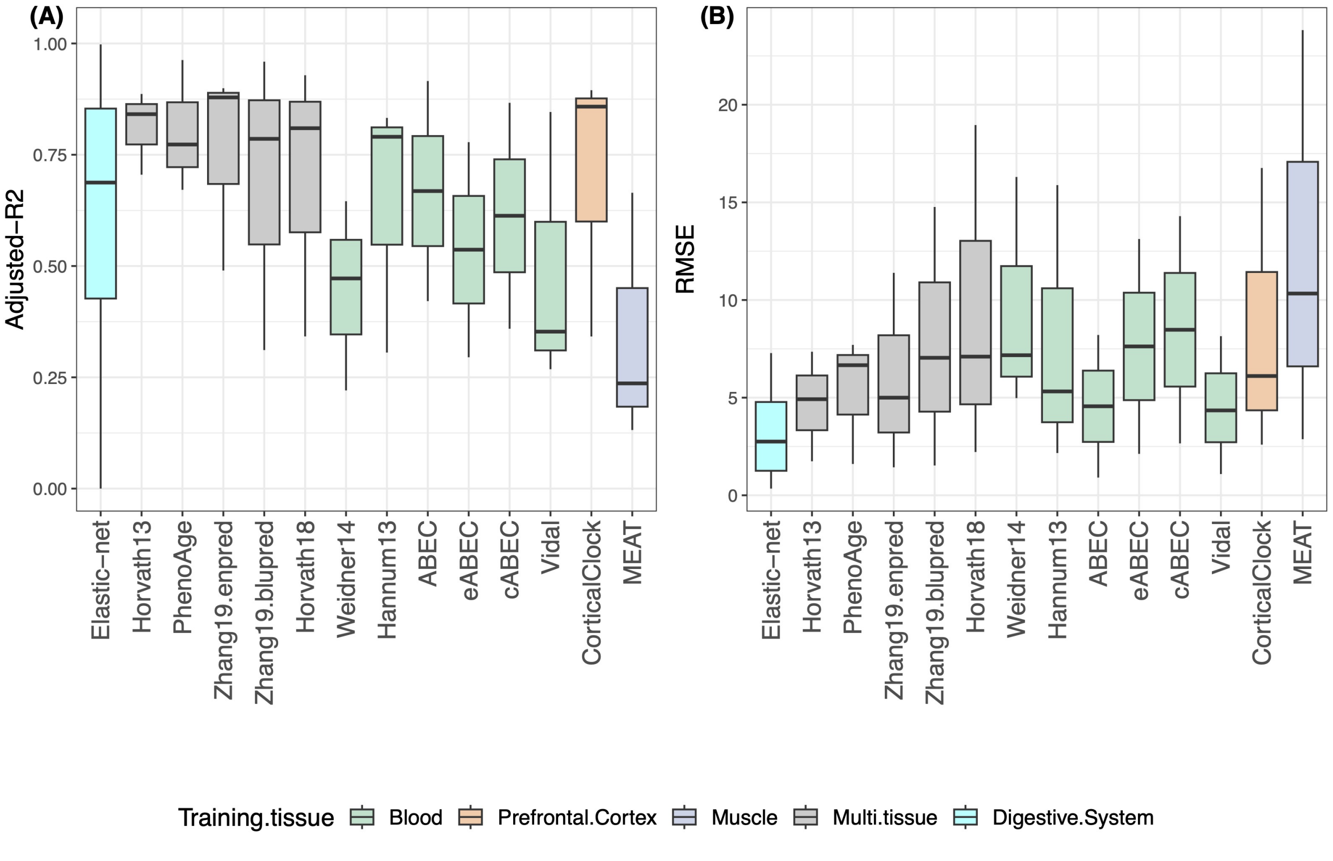

Figure S8. Comparison of the performance of different clocks in Liver. The box-plots areas are coloured according to the tissue of origin of the training set samples of each clock.

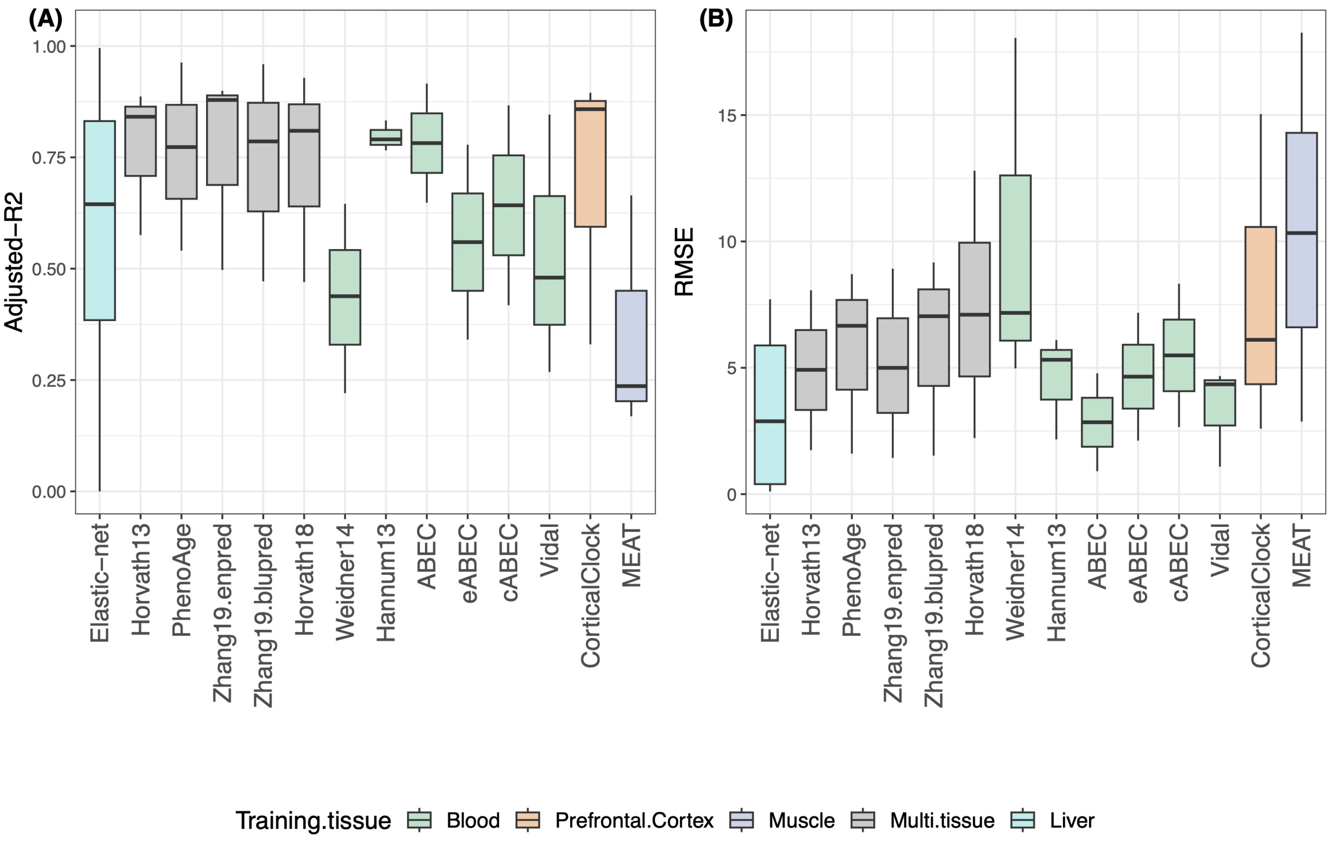

Figure S9. Comparison of the performance of different clocks in Prefrontal Cortex. The box-plots areas are coloured according to the tissue of origin of the training set samples of each clock.

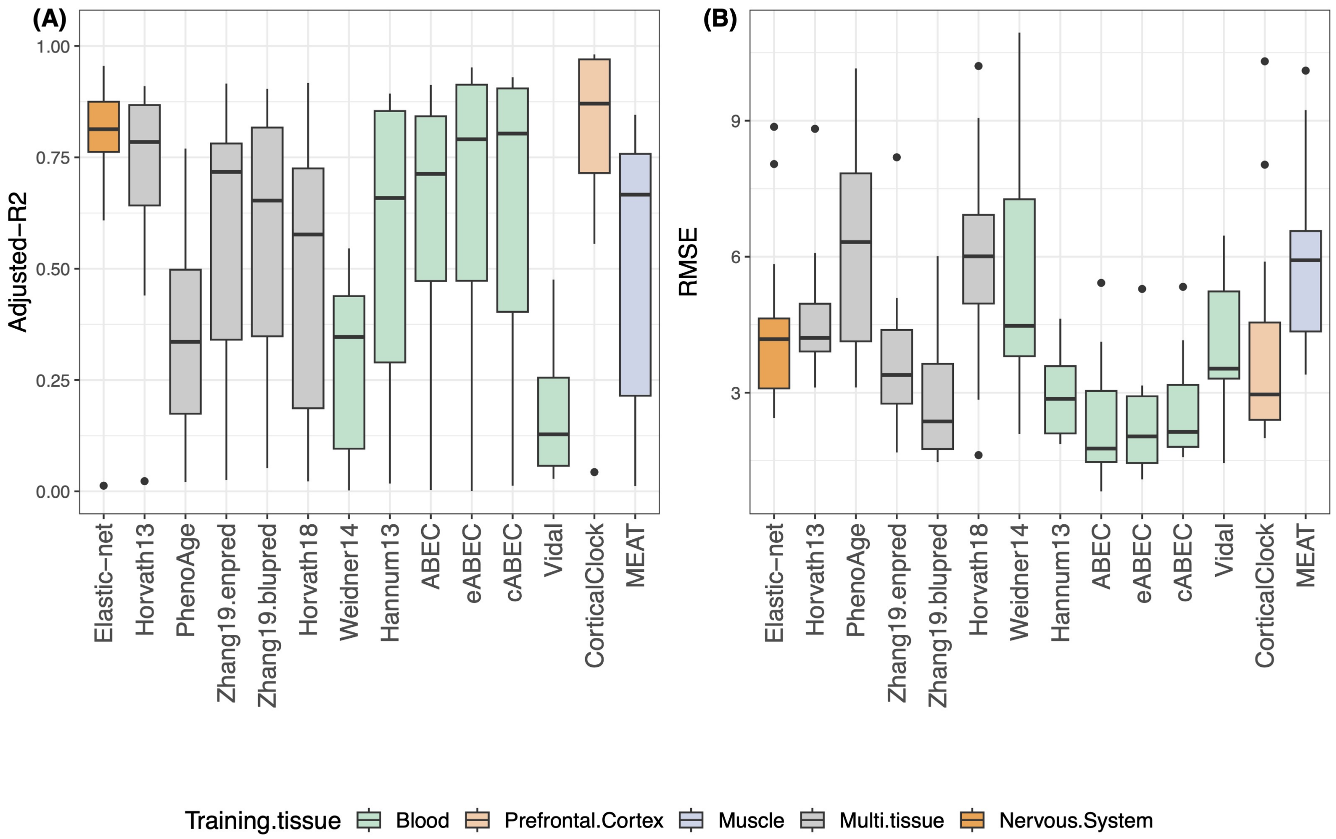

Figure S10. Comparison of the performance of different clocks in Whole Blood. The box-plots areas are coloured according to the tissue of origin of the training set samples of each clock.

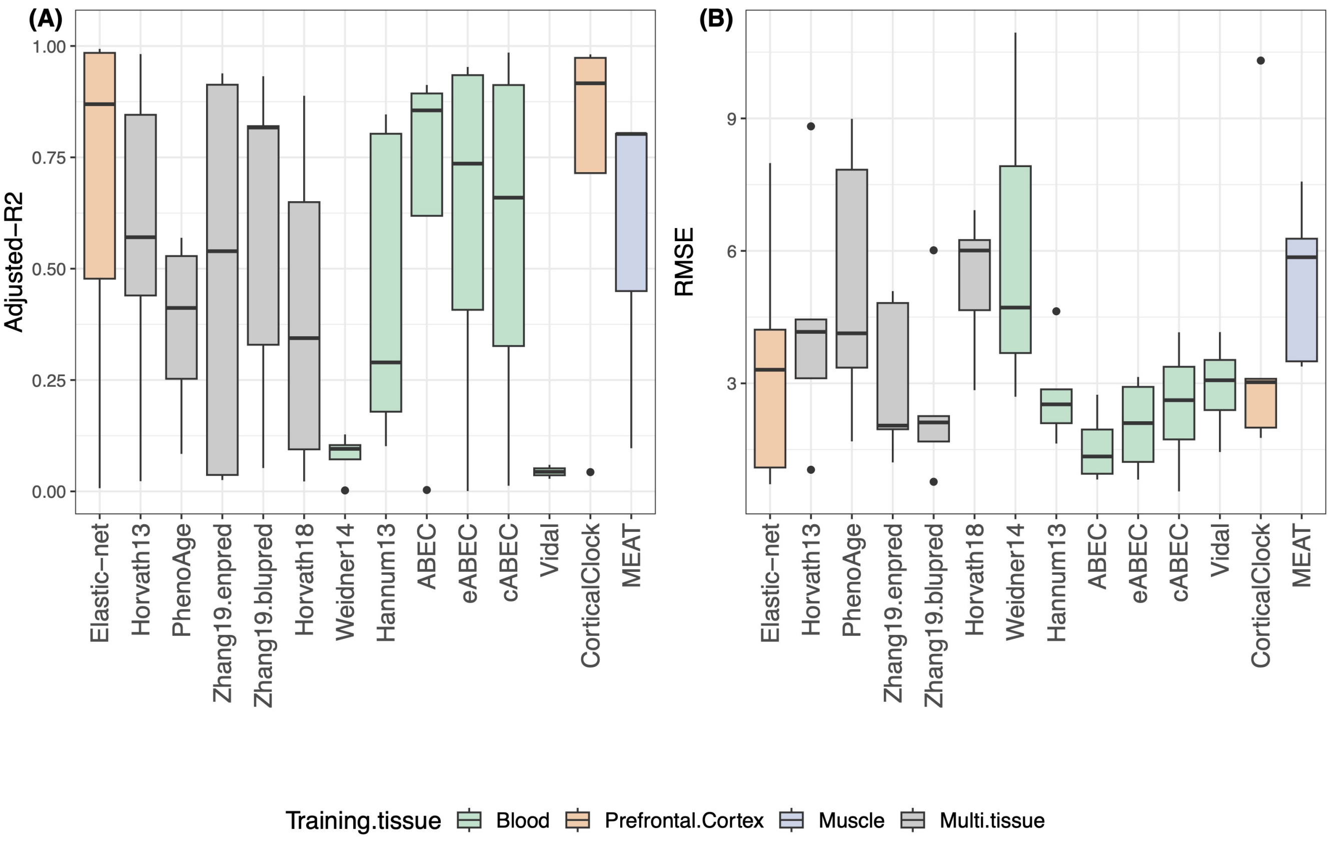

Figure S11. Comparison of the performance of different clocks in Digestive System. The box-plots areas are coloured according to the tissue of origin of the training set samples of each clock.

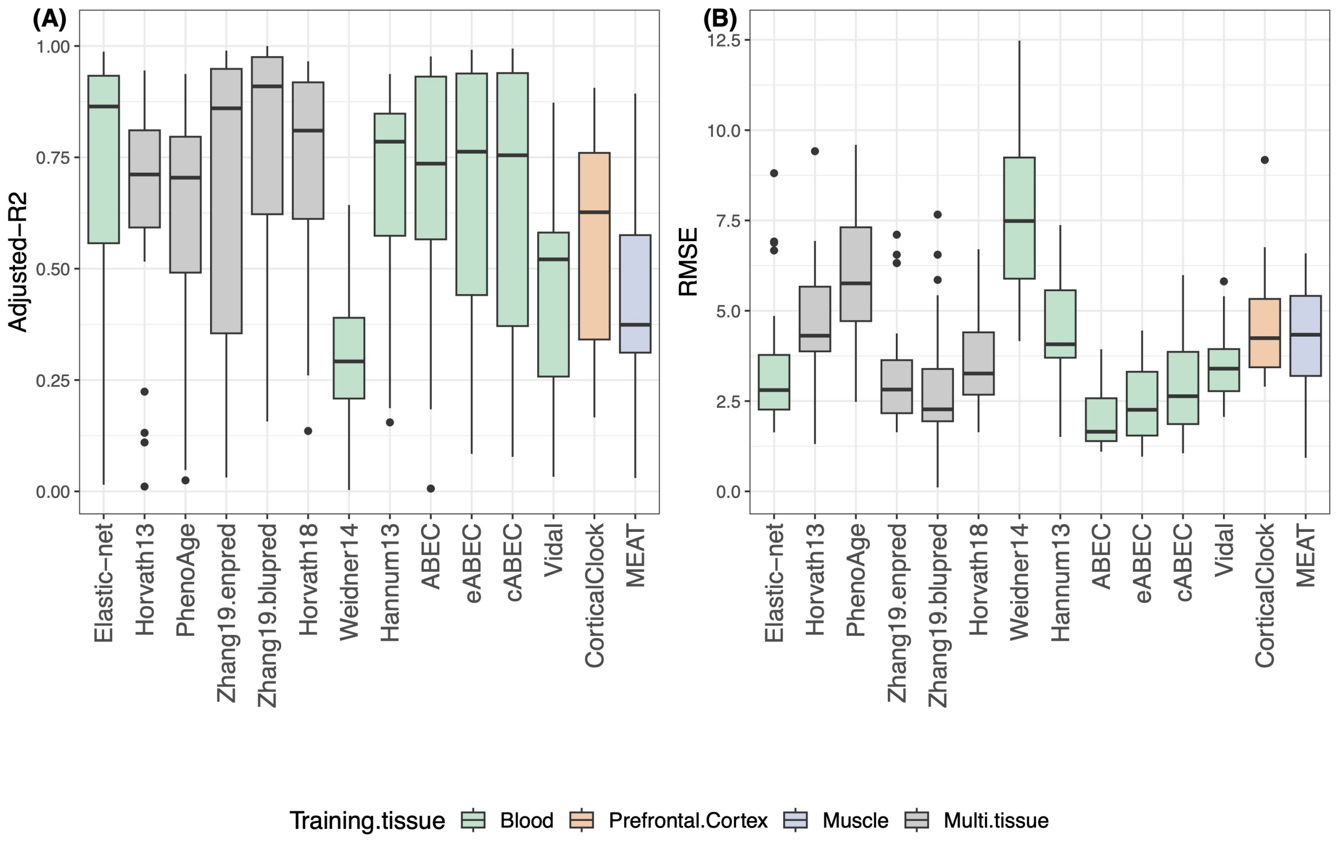

Figure S12. Comparison of the performance of different clocks in Nervous System. The box-plots areas are coloured according to the tissue of origin of the training set samples of each clock.

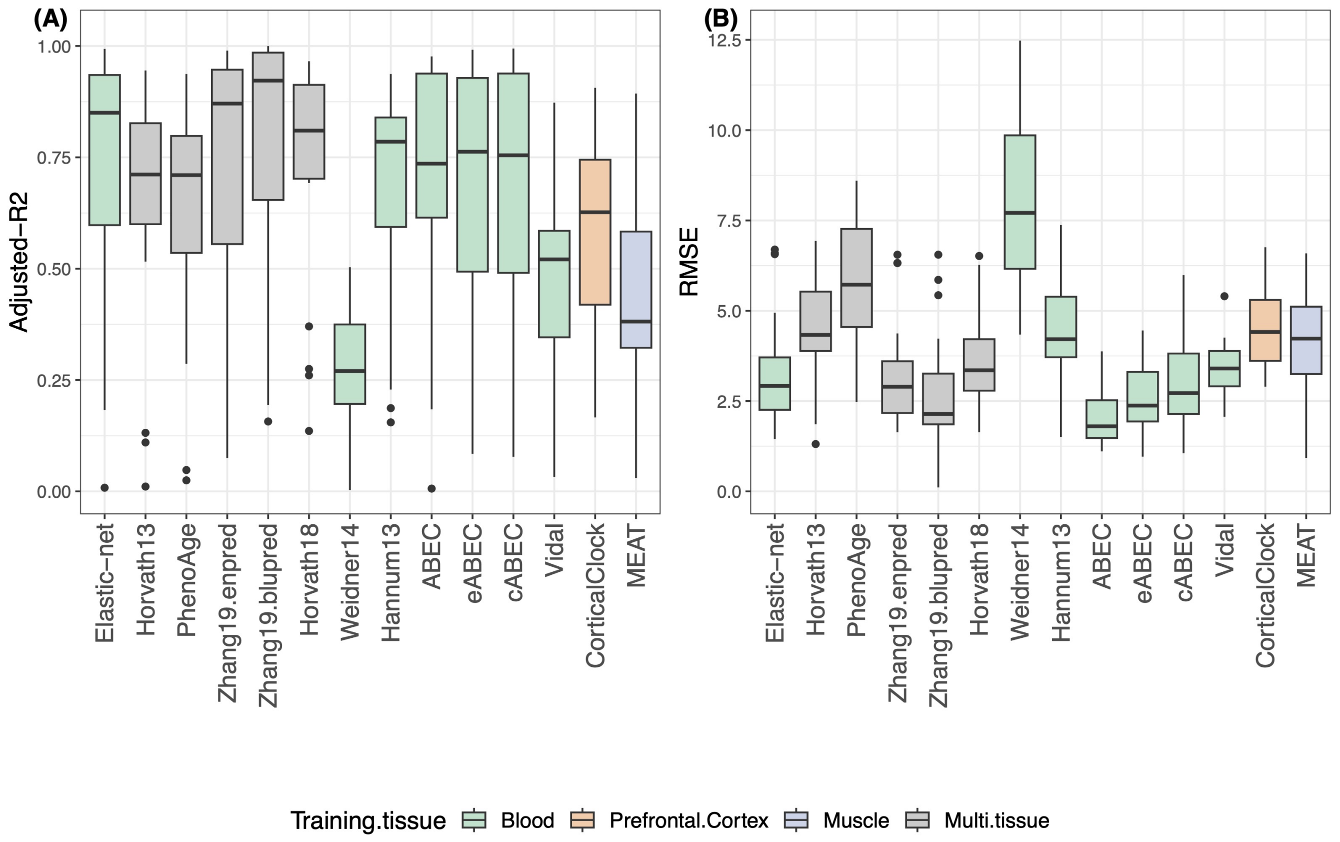

Figure S13. Comparison of the performance of different clocks in Blood. The box-plots areas are coloured according to the tissue of origin of the training set samples of each clock.

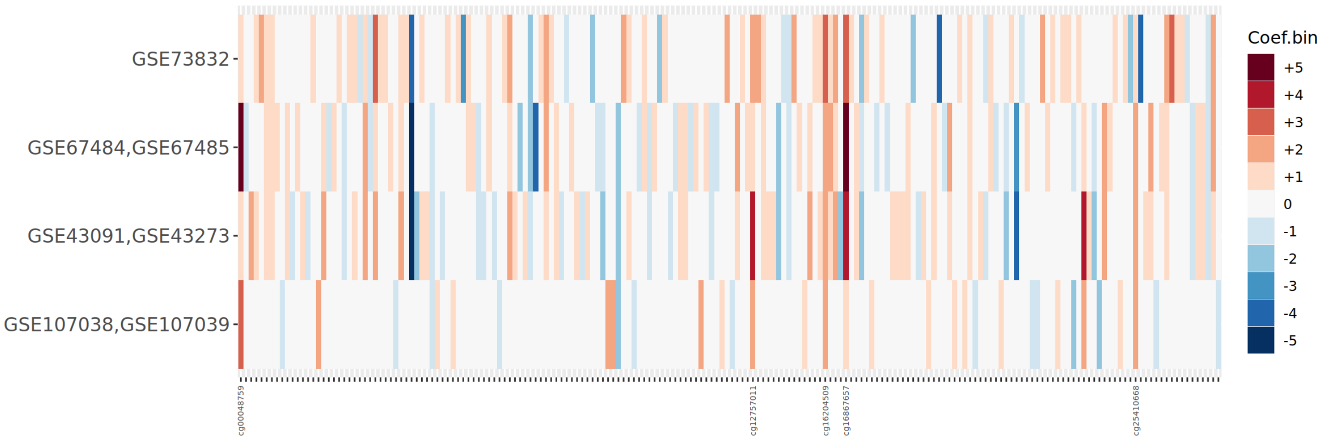

Figure S14. Heatmap of the partial effect of the selected CpGs in the Digestive System's clocks. The CpG's coefficients have been categorized to simplify the figure. On the x-axis only the CpGs with non-zero coefficients in all clocks are specified.

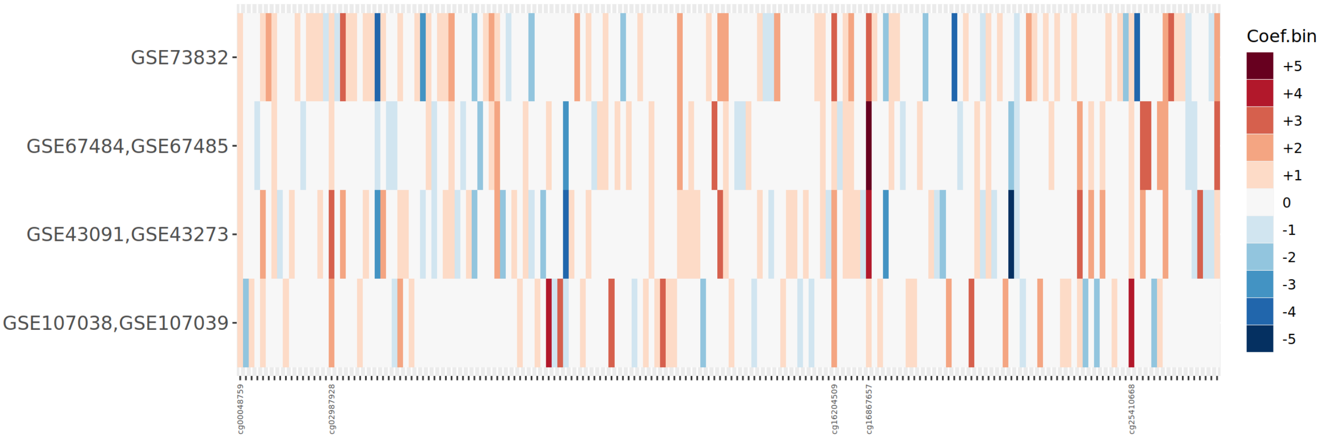

Figure S15. Heatmap of the partial effect of the selected CpGs in the Liver's clocks. The CpG's coefficients have been categorized to simplify the figure. On the x-axis only the CpGs with non-zero coefficients in all clocks are specified.

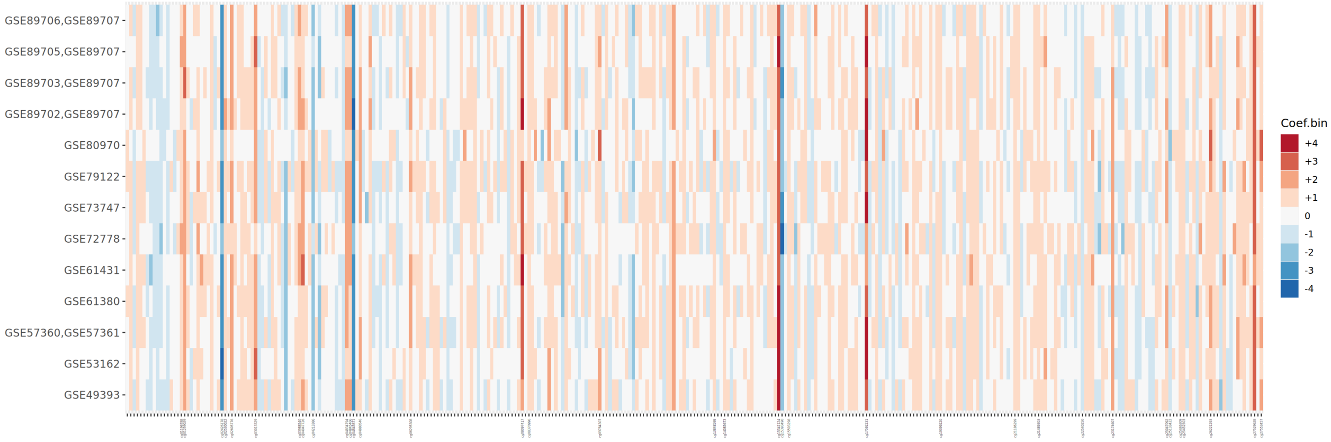

Figure S16. Heatmap of the partial effect of the selected CpGs in the Nervous System's clocks. The CpG's coefficients have been categorized to simplify the figure. On the x-axis only the CpGs with non-zero coefficients in all clocks are specified.

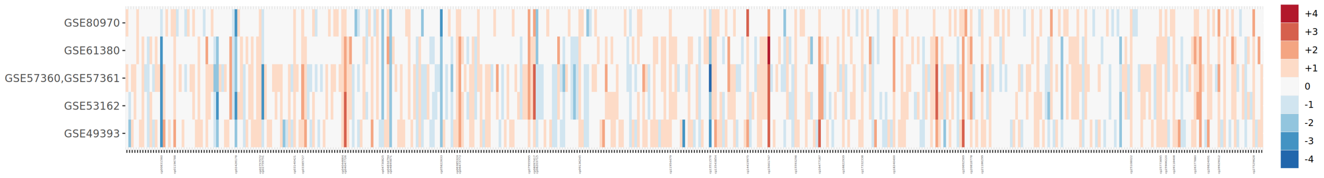

Figure S17. Heatmap of the partial effect of the selected CpGs in the Prefrotnal Cortex' clocks. The CpG's coefficients have been categorized to simplify the figure. On the x-axis only the CpGs with non-zero coefficients in all clocks are specified.

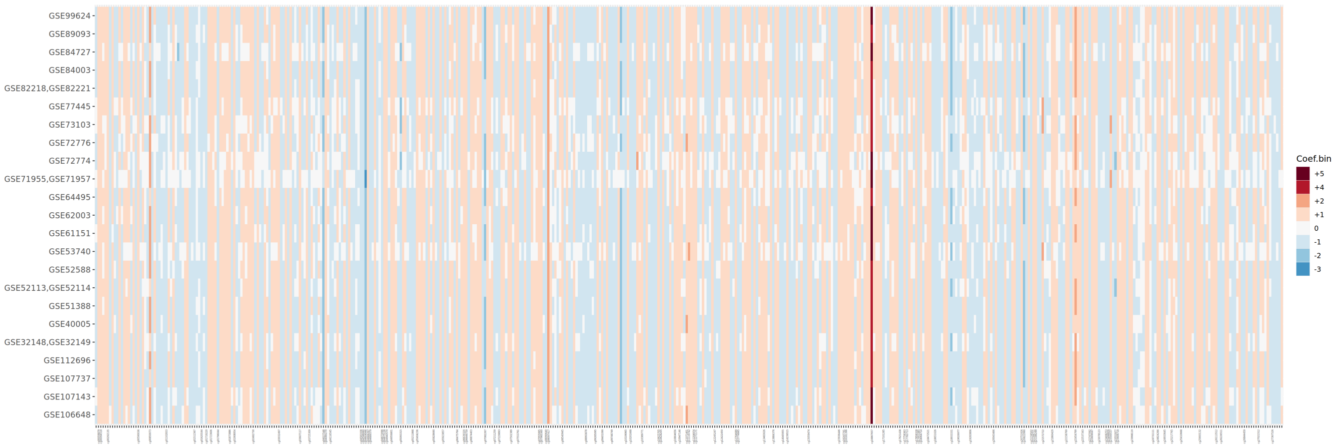

Figure S18. Heatmap of the partial effect of the selected CpGs in the Blood's clocks. The CpG's coefficients have been categorized to simplify the figure. On the x-axis only the CpGs with non-zero coefficients in all clocks are specified.

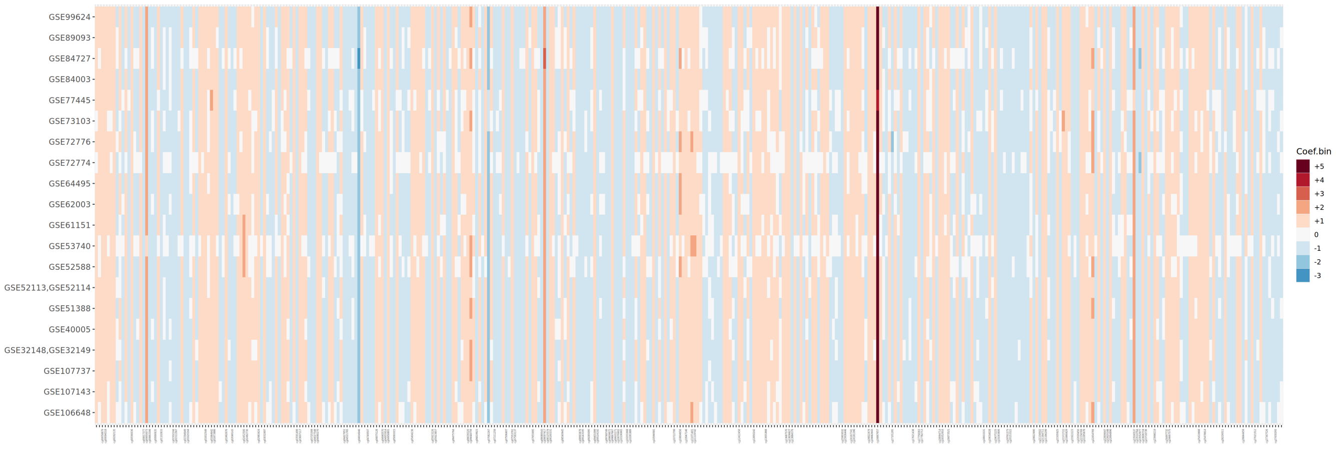

Figure S19. Heatmap of the partial effect of the selected CpGs in the Whole Blood's clocks. The CpG's coefficients have been categorized to simplify the figure. On the x-axis only the CpGs with non-zero coefficients in all clocks are specified.
